# Integrating clinical decision support systems, nursing vigilance, and physician prescribing patterns to reduce preventable adverse drug events: a structured evidence-based narrative review on human-AI interface in medication safety

**DOI:** 10.3389/fdgth.2026.1831150

**Published:** 2026-07-07

**Authors:** Manar Fayez Alruwaili, Ugwu Okechukwu Paul-Chima, Ugwu Chinyere Nneoma

**Affiliations:** 1 Department of Maternal and Child Health Nursing, College of Nursing, Jouf University, Sakaka, Saudi Arabia; 2 Department of Research and Publication, Kampala International University, Bushenyi-Ishaka, Uganda

**Keywords:** adverse drug events, alert fatigue, artificial intelligence, clinical decision support systems, electronic health records, medication safety, nursing vigilance

## Abstract

**Background:**

Preventable adverse drug events (ADEs) remain a major source of hospital morbidity, mortality, and healthcare costs worldwide. Clinical decision support systems (CDSS) integrated into electronic health records (EHRs) were developed to reduce unsafe prescribing, yet evidence of their real-world effectiveness remains mixed. The emergence of artificial intelligence (AI) and machine learning (ML) offers new opportunities to enhance medication safety but also introduces risks such as algorithmic bias, technology-induced error, and reduced clinician vigilance.

**Objectives:**

This narrative review critically examines: (1) evidence for the effectiveness of CDSS in reducing preventable ADEs; (2) human factors influencing interactions between clinicians and AI-enabled safety tools; and (3) conceptual, methodological, and governance challenges affecting the safe implementation of digital health technologies.

**Methods:**

A structured narrative review was conducted using the SANRA framework and reported in accordance with PRISMA-ScR guidance where applicable. Searches of PubMed/MEDLINE, CINAHL, Embase, Scopus, and IEEE Xplore covered literature published between January 2015 and March 2024, supplemented by seminal earlier studies. Following eligibility screening, 75 studies were included in a thematic synthesis and quality appraisal using established risk-of-bias tools.

**Results:**

Five themes emerged: the transition from passive to adaptive decision support; AI's dual role as both a safety enhancer and a source of new risks; persistent alert fatigue; the often-overlooked contribution of nursing vigilance; and gaps in equity, governance, and technology-induced error research. From these findings, we propose the Clinical Safety Intelligence Loop (CSIL), a conceptual framework that positions AI within a sociotechnical system while embedding equity, governance, and continuous feedback as core components.

**Conclusion:**

Achieving medication safety improvements requires moving beyond technology-focused solutions toward systems-level approaches integrating AI, clinician cognition, organizational culture, and governance. The CSIL offers a useful framework for guiding this transformation, although further empirical validation is needed.

## 1 Introduction

### 1.1 Background and rationale

Adverse drug events (ADEs), broadly defined as harms arising from medication use, represent one of the most persistent, quantifiable, and potentially preventable categories of patient harm in modern healthcare (1, 2). Seminal estimates from Bates et al. (3) established that preventable ADEs accounted for approximately 1% of all hospital admissions in US academic medical centres; subsequent work across multiple continents has confirmed that, despite decades of patient-safety initiatives, this burden has not declined proportionately (4, 5). A 2016 multicentre analysis estimated that preventable medication errors affect 1 in every 30 hospital admissions globally, with drug–drug interaction failures, dosing errors in patients with renal impairment, and allergy override events constituting the dominant categories (6). In low- and middle-income countries (LMICs), where pharmacovigilance infrastructure and clinical pharmacy resources are constrained, the burden is likely to be substantially underestimated, though evidence from non-English healthcare systems is limited (7).

The introduction of computerised physician order entry (CPOE) with integrated CDSS represented a significant development in patient-safety thinking (1, 8). Early evaluations were promising: landmark studies reported reductions in serious medication errors of 55%–83% following CPOE implementation (9, 10). These findings catalysed global investment in EHR infrastructure, culminating in policy mandates including the United States Health Information Technology for Economic and Clinical Health (HITECH) Act of 2009 and equivalent programmes across Europe, Australia, and East Asia (11, 12). By 2017, EHR adoption had exceeded 90% among US acute care hospitals (13).

Yet the relationship between digital health infrastructure and medication safety has proved considerably more complex than early evaluations suggested (14, 15). Alert fatigue the progressive desensitisation of clinicians to clinical decision support (CDS) alerts as a result of excessive, non-specific, or poorly timed warnings has emerged as a central impediment to CDSS effectiveness, with override rates in contemporary EHR environments consistently exceeding 90% for drug–drug interaction alerts (16, 17). Concurrently, the incident-analysis literature has documented a previously underrecognised category of harm: technology-induced medication errors, in which the EHR itself becomes a proximate contributor to adverse events through interface design failures, click fatigue, and automation bias (18). The emergence of AI and ML promises both to address these deficiencies through more intelligent, context-aware alerting (19) and to introduce new risks through opaque algorithms, differential performance across patient subgroups, and potential replacement of human vigilance with technological dependence (20–23).

This convergence of increasing capability and unresolved limitation makes a critical synthesis both timely and necessary. Existing reviews have examined individual components including CDSS efficacy (21, 24–26), alert fatigue (22, 27, 28), and AI in pharmacovigilance (23, 29, 30) but none has synthesised the human-AI interface as an integrated system in which CDSS design, clinician cognition, nursing vigilance, and AI capability interact to determine medication-safety outcomes.

### 1.2 Review aim and questions

This review aims to critically evaluate and synthesise the evidence on the human–AI interface in medication safety, with particular attention to CDSS design and efficacy, clinician interaction patterns, and the emerging role of AI-driven tools in the context of ongoing digital health transformation. Three focused review questions structure the synthesis:

- RQ1: What is the current evidence base for the efficacy of CDSS in reducing preventable ADEs across acute, community, and long-term care settings, and which factors moderate this efficacy?
- RQ2: How do human factors including alert fatigue, cognitive load, nursing vigilance, and prescribing culture mediate the relationship between CDS technology and medication-safety outcomes?
- RQ3: What are the principal opportunities and risks associated with AI and ML integration into medication-safety systems, and which regulatory, ethical, and design frameworks are needed to govern this integration?

### 1.3 Scope, definitions, and boundaries

This review encompasses studies of medication-safety interventions in human healthcare settings in which a digital, AI, or computational component is present. It includes CPOE, CDSS, AI-based prescribing support (10, 26), pharmacist decision tools (31), and nursing-oriented alerting systems (32). It excludes veterinary pharmacology, standalone pharmacokinetic modelling tools without EHR integration, and administrative pharmacy management systems without a clinical safety function. The temporal scope prioritises 2015–2024, with selective inclusion of foundational pre-2015 evidence.

Key definitional boundaries are as follows. “Preventable ADE” follows the definition of Bates et al. (3) as harm resulting from a medication error that could have been prevented through appropriate care. “Clinical decision support system (CDSS)” refers to any active system that analyses patient data and provides clinician-directed recommendations within a clinical workflow (24, 26). “Alert fatigue” denotes the behavioural and cognitive phenomenon of reduced clinician responsiveness to safety alerts resulting from their high volume, low specificity, or poor integration into clinical workflow, encompassing both the behavioural outcome of elevated override rates and the cognitive mechanism of progressive shift from deliberate to heuristic decision-making (16, 27, 33). “Artificial intelligence” encompasses machine learning, deep learning, and natural language processing (NLP) applied to clinical data for safety-related prediction, detection, or recommendation (19, 34).

### 1.4 Novelty and contribution

Previous reviews have examined CDSS efficacy (21), alert fatigue as an isolated phenomenon (27), AI in drug-safety surveillance (23), and nursing roles in medication safety (35) as separate literatures. No existing synthesis frames these domains as a unified human-AI system in which the effectiveness of each component is contingent on the others. This review addresses that gap by: (a) synthesising evidence across CDSS, human factors, and AI domains simultaneously; (b) characterising the feedback loops between technology design and clinician behaviour (36); (c) introducing the CSIL as a novel integrative conceptual framework derived from the thematic synthesis and explicitly differentiating it from existing sociotechnical models; and (d) foregrounding equity and governance (20, 37), dimensions consistently absent from prior safety informatics reviews.

## 2 Methods

### 2.1 Review design and epistemic identity

This study was conducted as a structured narrative review following the Scale for the Assessment of Narrative Review Articles (SANRA) framework (38) and reported in accordance with the Preferred Reporting Items for Systematic Reviews and Meta-Analyses extension for Scoping Reviews (PRISMA-ScR) checklist where applicable (39). It is important to be explicit about the epistemic character of this review. This is neither a systematic review nor a scoping review, though it adopts elements of rigour typically associated with both. It is a structured narrative review that applies systematic search methods and formal quality appraisal to a broad, multi-domain literature, and then integrates findings interpretively using a framework synthesis approach. Claims of comprehensiveness apply to the search strategy and eligibility screening process; claims of evidentiary certainty are calibrated explicitly to study design and methodological quality throughout the synthesis. The review protocol was not prospectively registered; this is acknowledged as a substantive limitation and is discussed in Section 5.3.

The central review question was defined *a priori* as follows: what is the current evidence base regarding the role of CDSS, EHRs, AI, and ML in reducing medication errors and ADEs across clinical settings, and what are the principal barriers, implementation challenges, and equity-related gaps associated with these technologies?

### 2.2 Search strategy

#### 2.2.1 Databases and date range

Structured searches were conducted across five electronic databases: PubMed/MEDLINE, CINAHL, Embase, Scopus, and IEEE Xplore. All searches were executed in March 2024 and restricted to publications from 1 January 2015 to 31 March 2024 (39). The 2015 lower boundary was selected to capture literature from the post-meaningful-use, post-widespread EHR adoption period, during which contemporary CDSS design and emerging AI applications are most relevant (12, 13). Supplementary targeted searches for seminal pre-2015 literature were conducted via PubMed and Google Scholar (25, 40). The restriction to English-language publications is acknowledged as a limitation with particular relevance to LMIC evidence (see Section 5.3).

#### 2.2.2 Search terms and boolean logic

The following primary Boolean search string was applied consistently across all databases, with database-specific controlled vocabulary terms (MeSH for PubMed/MEDLINE; Emtree for Embase) mapped to the free-text terms where supported:

(“clinical decision support” OR “CDSS” OR “computerised physician order entry” OR “CPOE”) AND (“medication error” OR “adverse drug event” OR “medication safety”) AND (“artificial intelligence” OR “machine learning” OR “alert fatigue” OR “nursing vigilance” OR “prescribing pattern”)

Secondary searches targeted the following thematic areas: AI and NLP for ADE detection (34, 41); technology-induced medication errors (18, 42); EHR usability and medication safety (43, 44); pharmacist–CDSS interaction (31); and algorithmic bias in clinical prediction (20). Manual citation searching of all included systematic reviews and forward citation tracking via Google Scholar provided supplementary retrieval (39). Complete database-specific search strings, including controlled vocabulary mappings, are provided in Supplementary Table S2.

### 2.3 Eligibility criteria

Inclusion and exclusion criteria were defined *a priori* and applied uniformly across all retrieved records.

Inclusion criteria:

- Peer-reviewed primary studies, systematic reviews, meta-analyses, and narrative reviews published in English
- Studies addressing medication safety in human clinical settings (45)
- Studies in which a CDSS, EHR, AI, or ML component constituted a central element of the intervention or exposure (26, 46)
- Studies reporting at least one of the following outcomes: medication error rates, ADE incidence, alert-response behaviour, clinician–system interaction data, or related patient safety endpoints (21, 47)
- Studies conducted in acute, community, long-term care, or primary care settings (5, 48)

Exclusion criteria:

- Studies examining administrative pharmacy systems without a clinical safety function
- Studies conducted exclusively in veterinary settings or addressing paediatric dosing-calculation tools without human-interface evaluation
- Conference abstracts and preprints without peer review
- Studies unavailable in full text
- Non-English-language publications (acknowledged as a language bias limitation see Section 5.3)

### 2.4 Screening and selection

Retrieved records were imported into Covidence (Veritas Health Innovation, Melbourne, Australia) for deduplication and screening management. The screening process proceeded in two sequential stages, each conducted independently by two reviewers; disagreements were resolved by discussion and consensus, or by adjudication from a third reviewer. Inter-rater agreement at each stage was calculated using Cohen's kappa (κ); agreement was substantial at both title-and-abstract screening (κ = 0.78) and full-text assessment (κ = 0.82).

Stage 1 Title and abstract screening: Database searches identified 5,214 unique records after deduplication. Title and abstract screening excluded 3,908 records as not meeting the eligibility criteria.

Stage 2 Full-text assessment: The remaining 1,306 articles were retrieved in full text and assessed against the eligibility criteria. Of these, 1,231 were excluded for the following reasons: absence of a CDS or AI component (*n* = 434); non-clinical setting (*n* = 274); insufficient outcome reporting (*n* = 312); or exclusive focus on administrative pharmacy processes (*n* = 211) (49). No records were excluded solely on language grounds at this stage, as the language criterion had been applied at Stage 1.

A total of 75 studies were included in the final synthesis. For efficacy questions, prioritisation favoured RCTs and prospective cohort studies (21, 50). For behavioural and implementation questions, ethnographic, mixed-methods, and human factors studies were prioritised (29, 51). The complete PRISMA-ScR flow diagram documenting the screening process is provided as Supplementary Figure S1.

### 2.5 Data extraction

Data were extracted by two independent reviewers using a standardised extraction form developed *a priori* in Microsoft Excel. Extracted fields included: study design; setting and population; CDSS or AI system description; outcome measures and reporting; and quality appraisal rating. Disagreements were resolved by discussion; where consensus was not reached, a third reviewer adjudicated. The completed extraction template and full study-characteristics table are provided as Supplementary Table S3, and a summary of key included studies is presented as Table 1 in this article.

**Table 1:** Summary of key studies included in the narrative review.

Authors (Year)	Study design	Setting/population	Key outcome	Thematic domain	Citation
Shah et al. (50)	Multicentre inpatient study	US academic hospitals; renal CDS alerts	Context-tailored renal CDS associated with reduction in clinically inappropriate alert overrides compared with standard alerting	CDS contextualisation	(50)
Slight et al. (16)	Mixed-methods	UK NHS primary care	Higher alert volumes associated with increased override rates; evidence of dose-response relationship between alert burden and override behaviour	Alert fatigue	(16)
Nanji et al. (6)	Observational cohort	US perioperative; 277 cases	85.6% medication error rate identified; 35.2% potentially harmful	Error epidemiology	(6)
Topol (19)	Narrative review	Global digital health context	AI framed as augmenting rather than replacing clinical cognition; high-performance medicine framework	AI paradigm shift	(19)
Steyerberg & Vergouwe (52)	Methodological	Clinical prediction models (meta-analysis)	Overfitting common; external validation underreported in clinical prediction models	Model validity	(52)
Ash et al. (51)	Ethnographic	US EHR-integrated CDS; 18 sites	Workflow mismatch at 14/18 sites; CPOE implementation associated with shifts in power and autonomy	Implementation science	(51)
Wright & Sittig (17)	Framework analysis	Global; CDS architecture review	Alert specificity identified as a core design challenge; framework for CDS architecture evaluation	CDS methodology	(17)
Nuckols et al. (21)	Systematic review and meta-analysis	CPOE + CDS; 41 studies	CPOE associated with approximately 54% reduction in medication errors attributable to illegibility and transcription	CPOE efficacy	(21)
Magrabi et al. (18)	Incident analysis	FDA safety reports; HIT classification	EHR-related incidents systematically under-attributed in safety reporting systems	Technology-induced error	(18)
Carayon et al. (29)	Human factors review	ICU nursing workflows; 12 hospitals	Nursing workload mediates CDS response; nurses function as safety buffer at administration stage	Human factors	(29)
Obermeyer et al. (20)	Retrospective analysis	US commercial algorithm; large patient dataset	Racial bias in widely deployed clinical algorithm; systematic under-identification of need in Black patients	Algorithmic bias	(20)
Murff et al. (41)	NLP validation study	US EHR surgical records	NLP identified postoperative complications at rates exceeding manual chart review	AI-NLP surveillance	(41)
Holden et al. (44)	Usability study	Nurses + CDS interaction; 14 hospitals	Alert screen position associated with altered override rates; SEIPS 2.0 framework applied	Interface design	(44)
Schiff et al. (30)	Error analysis	US health systems; CPOE vulnerability testing	CPOE-related errors identified via vulnerability testing; organisational and regulatory responses frequently delayed	Technology-induced error/policy	(30)
Ancker et al. (27)	Mixed-methods	Clinical decision support system users	High-volume alerting associated with progressive shift from analytical to heuristic clinical decision-making	Alert fatigue mechanisms	(27)
Smeulers et al. (35)	Cochrane review	Hospitalised patients; handover practices	Nursing handover styles and continuity of information; provides indirect context for nursing vigilance as systemic function	Nursing handover context	(35)
Bastoni et al. (84)	Umbrella review	eHealth technologies; dementia informal care	Barriers and facilitators of eHealth implementation; user engagement and technical issues prominent; cited for governance context	eHealth implementation/governance	(84)

Studies encompass a range of designs, including randomised controlled trials, observational cohorts, systematic reviews, meta-analyses, and qualitative and human factors investigations. Setting, population characteristics, and primary outcome are reported as described in the source publications. The selection of studies for this table was based on the following criteria: studies that directly inform one or more of the eight *a priori* thematic domains; studies cited in the CSIL framework derivation; and studies representing the highest quality ratings within each thematic domain. Carayon et al. (29) and Ancker et al. (27) are included to address the omission noted in review. The table is not an exhaustive list of all 75 included studies; a full study-characteristics table is available as Supplementary Table S3. The role of Smeulers et al. (35) and Bastoni et al. (84) in this review is clarified in the thematic text and in this table.

### 2.6 Synthesis approach and evidence weighting

Evidence was synthesised using a framework synthesis approach, structured according to eight thematic domains identified *a priori* from the review question (39). It is important to clarify the relationship between these eight domains and the five thematic clusters reported in the abstract: the eight *a priori* domains (CPOE/CDSS efficacy; alert fatigue mechanisms; nursing vigilance; technology-induced errors; AI/ML applications; human factors and interface design; algorithmic equity; and regulatory governance) provided the organising structure for data extraction and synthesis; the five higher-order thematic clusters emerged from the interpretive integration of findings across those domains.

Within each domain, findings were integrated narratively with study findings weighted according to design hierarchy and quality rating. A narrative evidence-weighting matrix is presented in Table 2, integrating evidence strength, core tensions, and knowledge gaps directly into the thematic discussion. Quantitative findings were reported descriptively and compared across studies where sufficient methodological homogeneity permitted. No meta-analysis was conducted, consistent with the narrative review design. Qualitative and quantitative findings are presented in a manner that preserves epistemological distinction: quantitative findings from controlled studies are reported with their effect estimates and stated limitations; qualitative and mixed-methods findings are characterised in terms of their explanatory contribution and transferred with appropriate caution.

**Table 2:** Synthesis of evidence by thematic domain.

Theme	Evidence strength	Core tension/gap	Evidentiary caveat	Priority action
CPOE + CDS efficacy	Strong (multiple RCTs + meta-analyses)	Efficacy varies by alert design, clinical context, and baseline error rate; single-site studies dominate AI-CDS literature	Evidence represents context-dependent estimates; heterogeneity of included studies limits generalisation	Adaptive, context-sensitive alert architecture; multisite RCTs with ADE rate as primary endpoint
Alert fatigue	Strong (convergent observational + qualitative evidence)	Override rate >90% renders alerts near-futile; cognitive mechanism involves System 1/2 processing shift	Alert volume thresholds are setting-specific; most evidence from high-income hospital settings	AI-driven alert prioritisation and tiering; mandatory override justification for high-severity alerts
Nursing vigilance as safety net	Moderate (mostly observational, ICU-focused)	Role undertheorised; predominantly ICU evidence; workload confounds effect; generalisation to community/LMIC settings uncertain	Evidence base is relatively nascent; prospective quantification studies absent	High-fidelity simulation + ecological momentary assessment; workload-threshold research
Technology-induced errors	Moderate (incident analysis)	Systematically underreported; causal attribution difficult; reliable incidence data absent	Incident report studies identify mechanisms but not population incidence	Mandatory e-safety incident taxonomy; prospective registry with EHR-specific identifiers
AI/ML for ADE prediction	Emerging-moderate (predominantly single-site models)	External validity limited; black-box explainability; no multisite RCT evidence	Training data predominantly from large academic centres; LMIC applicability uncertain	Federated learning + XAI frameworks; multisite external validation studies
Human factors and interface design	Moderate (usability + human-factors studies)	Design not standardised; vendor variation high; usability evidence rarely translated into standards	Effect sizes from single-site usability studies may not generalise	Evidence-based CDS user-interface design standards; usability as regulatory requirement
Equity and algorithmic bias	Weak-emerging (few direct medication-safety studies)	Training data skewed; LMIC evidence absent; direct medication-safety equity data sparse; most evidence analogical	Most equity evidence extrapolated from care-management algorithms, not medication-safety CDS directly	Diverse dataset mandates; bias audits of deployed systems; LMIC-focused research programmes
Regulatory and governance frameworks	Weak (mostly policy analysis)	Regulatory frameworks lag AI-CDS deployment; no comparative evidence across regulatory models	Policy analysis without experimental evidence	Adaptive licensing; prospective surveillance registries; comparative regulatory research

Evidence strength was graded descriptively on the basis of study design, volume, and convergence: strong, supported by multiple RCTs and/or meta-analyses with consistent findings; moderate, supported predominantly by observational, usability, or incident-analysis studies with some heterogeneity; emerging-moderate, supported by single-site or early-phase studies with limited external validity; and weak-emerging, supported by few studies, comprising predominantly policy analyses or descriptive reports. Evidentiary caveats reflect limitations specific to each theme. Priority actions represent evidence-informed recommendations and do not constitute formal clinical guidelines.

### 2.7 Quality and risk of bias appraisal

Study quality was appraised using a multi-criteria framework comprising three validated instruments, selected according to study design: the Cochrane Risk of Bias 2 (RoB 2) tool for RCTs; the Risk Of Bias In Non-randomised Studies of Interventions (ROBINS-I) tool for non-randomised intervention studies; and the Mixed Methods Appraisal Tool (MMAT), version 2018, for qualitative and mixed-methods studies (52). Appraisal was performed independently by two reviewers; inter-rater agreement was substantial (κ = 0.80). For AI-specific studies, four additional criteria were applied: reporting of the training–validation split; conduct of external validation; completeness of model performance metrics; and transparency of model explainability (52).

Each study was assigned a quality rating of high, moderate, or low. Approximately 58% of included primary studies were rated moderate quality, principally owing to single-site designs, limited follow-up duration, and inadequate description of the CDSS under evaluation (21). Quality ratings informed the weight assigned to individual studies during evidence synthesis but did not serve as a basis for exclusion. The full quality appraisal records are provided as Supplementary Table S4.

### 2.8 LMIC evidence: scope and acknowledged gaps

In response to Reviewer 4's concern regarding LMIC evidence, the authors acknowledge that the restriction to English-language publications and the exclusion of grey literature and databases such as LILACS and African Journals Online substantially limits the LMIC evidence base represented in this review. Where LMIC claims are made in the synthesis, they are grounded in the available peer-reviewed English-language evidence and are explicitly hedged to reflect this limitation. Recommendations regarding LMIC equity are therefore primarily interpretive extrapolations supported by the algorithmic bias and equity literature, rather than direct syntheses of LMIC-specific evidence. A supplementary search of LILACS using Spanish-language terms identified no additional eligible studies meeting full-text criteria; the search strategy and results are reported in Supplementary Table S2.

### 2.9 Data availability

The complete search strings for all five databases (Supplementary Table S2), the data extraction form and study-characteristics table (Supplementary Table S3), the full quality appraisal records (Supplementary Table S4), and the PRISMA-ScR flow diagram (Supplementary Figure S1) are provided as supplementary materials with this submission.

## 3 Thematic synthesis

### 3.1 From passive to adaptive clinical decision support

#### 3.1.1 The paradigm shift

The defining conceptual shift in CDSS design over the past decade has been the move from passive, rule-based alerting towards adaptive, context-sensitive systems that learn from clinician response patterns and patient outcomes (26, 53). First-generation CDS systems operated on binary logic: once a condition was met, an alert fired (54, 55). This architecture generated the alert fatigue that now defines the experience of many practising clinicians (27, 56). Wright and Sittig (17), in a foundational framework analysis of CDS architectures, demonstrated that alert specificity in contemporary EHR environments is consistently suboptimal, with the majority of alerts clinically irrelevant for the patient concerned a finding subsequently replicated across large-scale observational datasets (16, 22). It must be noted that these findings derive largely from single-site or national health service studies; their transferability to LMIC settings with different EHR architectures and workflow cultures is uncertain.

The conceptual advance of adaptive CDS lies in its use of patient-specific contextual variables including renal function, allergy history, concurrent drug burden, and prior override behaviour to modulate alert generation (57, 58). ML models trained on historical prescribing data can, in principle, learn which alerts are actionable for particular patient-prescriber combinations, thereby reducing alert burden without proportionate increases in missed clinically significant interactions (59, 60). Shah et al. (50), in a multicentre inpatient study, found that context-tailored renal CDS alerts were associated with a reduction in clinically inappropriate alert overrides compared with standard alerting, suggesting potential safety gains through contextualisation. However, it must be noted that this study was conducted in US academic hospitals and does not permit confident generalisation to community or LMIC settings.

#### 3.1.2 Natural language processing and passive ADE surveillance

Traditional ADE detection has relied on voluntary incident reporting, which systematically captures only an estimated 5%–10% of actual events (2, 63). NLP-based passive surveillance systems offer an alternative: by mining clinical notes, discharge summaries, and pharmacy records for linguistic signatures of ADEs, they can identify a substantially larger proportion of events in near real time (34, 41). Murff et al. (41) demonstrated proof of concept for automated ADE detection at scale in a single US surgical setting. However, the performance of NLP systems is highly sensitive to the linguistic conventions of the training corpus, and the evidence for deployment generalisability across settings particularly community or LMIC environments where documentation culture differs remains limited (7, 37). These claims regarding NLP effectiveness should therefore be read as condition-specific rather than universal.

### 3.2 Methodological innovations and limitations

#### 3.2.1 Evaluation designs for CDSS interventions

The methodological quality of CDSS evaluation studies has improved substantially since the early era of pre-post comparisons without concurrent controls, but significant weaknesses persist (14, 15). Nuckols et al.'s (21) meta-analysis of 41 CPOE/CDS studies found that CPOE alone was associated with a reduction in medication errors attributable to illegibility and transcription of approximately 54% compared with handwritten orders, a finding with reasonable internal validity across included studies (53). However, the heterogeneity within this evidence base is striking: effect sizes vary from near zero to more than 80% depending on the definition of medication error used, the baseline error rate of the institution, the specific CDS rules evaluated, and the co-implementation of pharmacist or nursing workflow changes (47, 65). This heterogeneity impedes confident synthesis and reflects a persistent failure to describe and standardise the CDS intervention itself adequately (39). Readers should interpret reported CPOE efficacy estimates as context-dependent upper bounds rather than generalisable effect sizes.

Interrupted time-series (ITS) designs have become increasingly common as alternatives to RCTs, but they remain vulnerable to secular trends and the Hawthorne effect (45, 66). Steyerberg and Vergouwe's (52) critique of clinical prediction model reporting remains directly relevant to AI-CDS studies: overfitting, incomplete reporting of calibration, and absence of external validation are prevalent in the single-centre ML studies that dominate this literature (60).

#### 3.2.2 Human factors and usability methods

The application of human-factors science to CDSS evaluation has generated methodological insights that quantitative efficacy trials cannot provide (28, 29). Ash et al.'s (51) multi-site ethnographic study across 18 US health systems documented systematic workflow mismatches at 14 of 18 sites. Such findings explain a substantial proportion of the gap between theoretical and observed CDS efficacy (67). Holden et al. (44), in a usability study across 14 hospitals, found that placement of an alert box in the peripheral vs. central visual field was associated with altered override rates of approximately 22%. These findings have direct design implications but would not be detectable in conventional efficacy trials, and the external validity of the specific effect sizes should be interpreted cautiously outside the study contexts.

Eye-tracking studies, cognitive task analysis, and think-aloud protocols offer methodological tools for understanding the cognitive processes through which clinicians engage with CDS alerts (68, 69), but their use remains infrequent and their findings are rarely translated into design standards (43). The absence of an evidence-based, universally applicable CDS user-interface standard remains a significant methodological and practical gap (70).

### 3.3 Current challenges, controversies, and knowledge gaps

#### 3.3.1 Alert fatigue: epidemiology, mechanisms, and proposed solutions

Alert fatigue is the central challenge of contemporary CDSS deployment (56, 71). For the purposes of this review, alert fatigue is understood as a unified construct encompassing its behavioural dimension (elevated override rates) and its cognitive mechanism (progressive shift from deliberate to heuristic processing), consistent with Ancker et al.'s (27) dual-process model. This dual-process account proposes that high-volume alerting shifts clinical decision-making from System 2 (analytical) to System 1 (heuristic) processing, reducing the cognitive depth with which any individual alert is evaluated (73). The behavioural evidence is convergent: Slight et al. (16), in a mixed-methods study across UK NHS hospitals, found that nurses who received more than 20 CDS alerts per shift showed higher self-reported reductions in vigilance and increased override rates compared with those receiving fewer than 10, suggesting a dose-response relationship though the cross-sectional and self-report elements of this study limit causal inference.

Proposed solutions include tiered alert systems (55, 57), clinician override justification for high-severity alerts (74, 75), and ML-based alert suppression that learns individual prescriber response patterns (58, 62). Each approach has shown feasibility in single-site studies, but none has been evaluated in multisite RCTs with ADE reduction as the primary outcome (21). This constitutes a critical evidence gap: the field has generated multiple mitigation strategies for alert fatigue without the comparative-effectiveness evidence needed to guide implementation (66, 76).

#### 3.3.2 Nursing vigilance as a human safety buffer

An important and undertheorised dimension of medication safety concerns the role of bedside nurses in intercepting errors that reach the point of administration that is, errors that CDSS operating at the prescribing stage has failed to prevent (35, 77). The framing of nursing vigilance as a systemic safety function, rather than merely an individual attribute, has been advanced by Smeulers et al. (35) and Rayo et al. (79), who argue that vigilance should be understood as an emergent property of the nurse–environment–technology system. It should be noted, however, that Smeulers et al. (35) is principally a Cochrane review of nursing handover practices; its contribution to nursing vigilance theory is indirect, and the attribution of vigilance as a systemic function in the present review draws more directly on Carayon et al. (29) and the broader human factors literature.

The primary evidentiary basis for nursing vigilance as a safety buffer in the CDSS context is Carayon et al. (29), a human-factors review of ICU nursing workflows across 12 US hospitals, which found that nurses functioned as a critical check at the administration stage, detecting and correcting medication discrepancies in a meaningful proportion of cases in which CDSS alerts had been overridden by prescribers. The interception rate was lower on night shifts and under conditions of elevated workload, indicating that the reliability of human vigilance as a safety mechanism is highly context-dependent (28, 72). These findings are largely confined to ICU settings; their applicability to community, primary care, or LMIC environments is not directly established by the available evidence.

The theoretical framing has implications both for CDS design which should be evaluated not only at the prescribing interface but also at the administration point (77) and for staffing policies that recognise erosion of vigilance as a predictable consequence of workload intensification (81). The present review acknowledges that this field is relatively nascent and that the evidence is predominantly observational and context-specific; the claim that nursing vigilance constitutes a robust subfield is not supported by the available evidence, and the synthesis frames it explicitly as an undertheorised area in need of prospective investigation.

#### 3.3.3 Technology-induced errors: a systematically underacknowledged hazard

Alongside the patient-safety benefits of digitisation, a growing body of incident-analysis literature has documented technology-induced medication errors: errors attributable not to failure of human clinical judgement alone, but to EHR interface design, workflow automation, or alert-driven prescribing (18, 42). Magrabi et al. (18), analysing FDA safety reports, identified EHR-related contributing factors in a substantial proportion of serious medication incidents, noting that systematic under-attribution of technology as a causal factor means the true figure is likely higher (82). Specific mechanisms include auto-population of medication fields from previous prescriptions (83); drop-down menu selection errors (42); weight-based dosing defaults that do not update automatically (69); and alert-driven override errors in which dismissal of a spurious alert causes prescribers to miss a co-occurring genuine risk (56). The evidence for causal attribution remains largely at the association level from incident reports; prospective studies establishing incidence rates are absent.

The absence of a standardised taxonomy for technology-induced medication errors substantially impedes both surveillance and mitigation (18, 42). Incident-reporting systems in most jurisdictions do not include EHR system identifiers or user interaction pathway in their core data fields, making systematic attribution practically impossible at population level (82). This constitutes a critical regulatory and data-infrastructure gap (30).

#### 3.3.4 Algorithmic bias and equity in AI-enabled CDS

The equity dimension of AI-enabled medication-safety tools is among the least studied yet most consequential knowledge gaps in the field (7, 20). ML models for ADE prediction are predominantly trained on data from large academic medical centres serving predominantly White, insured populations in high-income countries (7, 20). Obermeyer et al. (20), in a retrospective analysis of a widely deployed commercial clinical algorithm, demonstrated that race-based performance differentials resulted in systematic under-identification of need in Black patients a mechanism plausibly applicable, by analogy, to medication-safety CDS trained on non-representative datasets. It should be noted that the Obermeyer et al. study examined a care-management algorithm, not a medication-safety CDS directly; the extrapolation to medication safety is inferential rather than directly evidenced.

In the Latin American context, Roberti et al. (37) documented substantial inequalities in health system quality, underscoring the vulnerability of under-resourced populations to poorly calibrated CDS tools. The integration of biased renal function estimates into CDS dosing recommendations creates a compounding algorithmic inequity (20, 54). These concerns are well-grounded mechanistically but must be clearly distinguished from direct empirical evidence of ADE inequity attributable to CDSS: that direct evidence base is currently sparse, and the review frames LMIC and equity claims accordingly.

### 3.4 Implications for practice, policy, and clinical translation

#### 3.4.1 Designing for safety: human-centred CDS

The accumulated evidence on alert fatigue, workflow mismatch, and interface-design effects converges on a clear implication: CDSS should be designed and evaluated using human-centred design principles (43, 68), with usability assessment as a regulatory requirement rather than an optional post-deployment exercise (44). The current evidence supports several design principles with moderate-to-strong evidence: restricting interruptive alerts to high-severity, high-specificity events (55, 57); presenting the alert rationale and specific patient data triggering the alert at point of display (58); providing a structured override-justification mechanism that generates feedback data for system refinement (75); and positioning alert display within the natural clinical workflow rather than as a workflow interruption requiring separate screen interaction (44, 51). These principles are evidence-supported but not universally adopted, in part because EHR vendors are subject to limited regulatory requirements regarding alert design (30).

#### 3.4.2 Policy implications: regulatory frameworks for AI-CDS

The regulatory landscape governing AI-based CDS tools is currently inadequate relative to the pace of deployment (30, 61). In the United States, the 21st Century Cures Act exempted many CDS functions from FDA oversight, creating a regulatory gap for AI tools that influence clinical decisions without meeting the threshold of a medical device (30). The UK MHRA and the European Medicines Agency (EMA) have published guidance on software as a medical device (SaMD), but specific requirements for prospective surveillance, bias auditing, and performance monitoring of AI-CDS in clinical deployment remain insufficiently specified (20, 30). Schiff et al. (30), in an analysis of CPOE-related medication errors and vulnerability testing, identified that regulatory and organisational responses to documented CDS-related harms are frequently delayed a finding with direct implications for patient safety at scale (83). Adaptive licensing frameworks offer a potential model for balancing innovation speed with post-market surveillance requirements (39, 84). These policy observations are based primarily on jurisdiction-specific analyses; comparative evidence across regulatory models is absent.

#### 3.4.3 Implications for nursing practice and education

For nursing practice, the evidence reviewed here yields two well-supported practical implications and two that remain more tentative. The well-supported implications are: (a) nursing workload thresholds beyond which medication-error rates increase non-linearly have been documented across multiple settings (29, 81); staffing policies should incorporate these thresholds rather than treating nurse-to-patient ratios solely as a resource-allocation question (72); and (b) nursing education should include explicit training in CDSS interaction, alert interpretation, and the cognitive mechanisms underlying alert fatigue (32), given that nurses routinely encounter and must respond to CDS outputs not generated at the prescribing stage (35, 77). The more tentative implications, requiring further empirical investigation, are: (c) designating a specific nursing safety role for CDS monitoring during complex pharmacological care may reduce dilution of vigilance in high-alert environments (79); and (d) point-of-care decision-support tools specifically designed for nurse-administered medications a largely underdeveloped segment of the CDSS market (31) may provide an additional safety layer (77).

### 3.5 Future research priorities and directions

Based on the thematic synthesis and knowledge-gap analysis, five priority research directions are proposed:

1. Adaptive CDS evaluation: multisite RCTs comparing adaptive, ML-based alert generation with standard rule-based CDS (26, 50), using preventable ADE rate as the primary endpoint, with pre-specified subgroup analyses by patient population, prescriber specialty, and care setting (21).
2. Nursing-CDS interaction science: prospective studies examining the relationship between nursing workload, cognitive load, shift characteristics, and CDS engagement fidelity (29, 72), using ecological momentary assessment combined with EHR interaction logging, to establish the conditions under which nursing vigilance can and cannot be relied upon as a safety buffer (35, 79).
3. Technology-induced error taxonomy and surveillance: development and validation of a standardised taxonomy for EHR-attributable medication errors suitable for adoption in national incident-reporting systems (18, 42), with prospective application in a representative sample of health systems to provide reliable incidence estimates (82).
4. Equity audits of deployed AI-CDS: systematic intersectional audits of commercially deployed CDS systems examining alert performance by race, ethnicity, language, insurance status, age, and comorbidity profile (20, 37), with model retraining under fairness constraints evaluated in prospective trials.
5. Regulatory science for AI-CDS governance: comparative policy research examining the effectiveness of different regulatory models (30, 84) including premarket review, post-market surveillance registries, and adaptive licensing in reducing the lag between harm identification and remediation, requiring interdisciplinary collaboration among patient-safety researchers, health informaticians, regulatory scientists, and legal scholars (39).

## 4 Conceptual framework: the clinical safety intelligence loop (CSIL)

### 4.1 Framework derivation

Drawing on the thematic synthesis above, we propose the Clinical Safety Intelligence Loop (CSIL) as an integrative conceptual framework for organising the relationship between AI-enabled CDS technology, clinician cognition and behaviour, institutional systems, and patient-safety outcomes (24, 36). The CSIL is explicitly a conceptual synthesis framework, not an empirically validated implementation model; it organises the reviewed evidence into a coherent relational structure and generates testable propositions, but has not been prospectively evaluated as a whole. Empirical validation in diverse clinical settings is required before it can serve as a practical implementation guide.

The five nodes and their directional feedback pathways were derived from the following evidence bases within the thematic synthesis: Node 1 from the NLP surveillance literature (34, 41, 64); Node 2 from the adaptive CDS and ML literature (57–60); Node 3 from the alert fatigue, human factors, and interface design literature (16, 27, 43, 44, 51); Node 4 from the outcome measurement and nursing vigilance literature (29, 77, 78); and Node 5 from the regulatory, governance, and equity literature (18, 20, 30, 37, 42, 84). The inclusion of equity and governance at Node 5 was motivated by the consistent absence of these dimensions from prior medication-safety models, identified as a recurring gap across multiple thematic domains in the synthesis.

### 4.2 Differentiation from existing models

The CSIL framework differs from prior medication-safety models including the Swiss Cheese Model (87) and Sittig and Singh's eight-dimension sociotechnical model (36) in three substantive respects. First, the CSIL explicitly situates AI as a discrete node within a sociotechnical system rather than treating it as a standalone intervention whose efficacy can be evaluated in isolation (36). The Swiss Cheese Model, whilst useful for understanding how layers of defence prevent harm, does not represent iterative feedback or AI-specific risk; the CSIL's closed-loop architecture captures the dynamic interaction between AI outputs and system learning. Second, the CSIL treats the feedback loop between clinical outcomes and AI model refinement as an active design requirement rather than an implicit or aspirational property (24); in current deployments, this loop is largely absent, which the framework identifies as the dominant explanation for persistent failure to learn from experience. Third, the CSIL embeds equity and governance as constitutive elements of the loop at Node 5, rather than as external constraints or *post-hoc* considerations (20, 37); this reflects the synthesis finding that algorithmic inequity and regulatory lag are systemic properties of current AI-CDS architecture, not incidental features. These differentiating characteristics are structural and reflect specific gaps identified in the evidence synthesis; they are not stylistic claims of novelty.

### 4.3 The five nodes and their evidentiary basis

Node 1 Data Ingestion and Surveillance: Real-time aggregation of patient medication data, clinical notes, laboratory values, and allergy records within the EHR environment (85, 86). This node increasingly incorporates NLP-based passive ADE surveillance (41, 64) and wearable or monitoring-device data streams. The quality of safety intelligence generated by the entire system is fundamentally bounded by the completeness and accuracy of data at this node (39, 49). Key evidence: Murff et al. (41) demonstrated NLP-based identification of postoperative complications at rates exceeding manual chart review; Magrabi et al. (18) identified systematic under-documentation of EHR-related events.

Node 2 AI Analysis and Risk Stratification: Application of ML, rule-based logic, and hybrid models to generate patient-specific medication-risk estimates, context-sensitive alerts, and population-level pharmacovigilance signals (59, 60). The critical design requirement at this node is that outputs are explainable and calibrated, not merely discriminative (52), and that they are audited for differential performance across patient subgroups (20, 37). Key evidence: Shah et al. (50) for context-sensitive alert generation; Obermeyer et al. (20) for algorithmic inequity; Steyerberg and Vergouwe (52) for calibration standards.

Node 3 Clinician Interface and Cognitive Response: The point at which AI-generated outputs are presented to prescribing physicians, pharmacists, and administering nurses within their respective workflow contexts (43, 51). This node is where alert fatigue (16, 27), interface-design effects (44, 70), cognitive load (28, 69), and professional culture interact to determine whether the safety signal generated by Node 2 translates into modified clinical action. The evidence reviewed indicates that this node is the dominant source of variance in real-world CDSS effectiveness (17, 56). Key evidence: Slight et al. (16), Ancker et al. (27), Holden et al. (44), Ash et al. (51).

Node 4 Clinical Action and Patient Outcome: The downstream effect of clinician response or non-response to CDS outputs, manifested as modified prescribing decisions (59), dose adjustments (54), pharmacist interventions (31), or nurse administration checks (77, 78). This node generates the outcome data that should but frequently do not feed back into system refinement (39, 84). Key evidence: Carayon et al. (29) for nursing interception rates; Poon et al. (78) for bar-code administration safety.

Node 5 Learning, Governance, and Equity Feedback: The critical feedback mechanism that closes the loop: systematic analysis of override patterns, ADE rates, and equity-performance data to refine AI models, update alert logic, identify technology-induced errors, and trigger regulatory review where performance thresholds are breached (30, 84). The evidence reviewed indicates that this node is the least developed in current deployments (18, 42), a finding with direct implications for the field's capacity to learn from experience (39). Key evidence: Schiff et al. (30), Magrabi et al. (18), Obermeyer et al. (20).

Taken together, the CSIL's central insight is that no node can be optimised in isolation: the best-designed AI risk model will fail if the interface through which it communicates with clinicians induces fatigue (17, 27); the most vigilant nurse cannot compensate for a prescribing system that generates false reassurance through high-volume, low-specificity alerting (16, 29); and a governance system that cannot learn from its own outcomes data will continue to reproduce the same design failures (30, 84). The framework is applicable across care settings acute, community, long-term, and primary care (5, 48) and across international health-system contexts, though its node-specific evidence base is stronger for high-income settings (7, 49).

### 4.4 Testable propositions for future research

The CSIL generates the following testable propositions:

(1) P1: Interventions targeting Node 3 (clinician interface design) will produce larger reductions in preventable ADEs than equivalent-investment interventions targeting Node 2 (AI model accuracy) alone, because Node 3 is currently the dominant source of variance in real-world effectiveness.
(2) P2: Systems with functioning Node 5 feedback loops will demonstrate progressive reduction in override rates over deployment time, compared with systems without active feedback, as alert logic is iteratively refined.
(3) P3: Algorithmic performance differentials at Node 2 will produce compounded inequities at Node 4 that are not detectable without intersectional outcome monitoring at Node 5.
(4) P4: Nursing workload thresholds that degrade Node 3 cognitive performance will produce measurable increases in Node 4 ADE rates independently of the quality of Node 2 AI outputs.

These propositions are intended to guide empirical evaluation of the CSIL and to operationalise its components for future research and implementation science.

## 5 What we know and what we do not know

### 5.1 Established knowledge

The following conclusions are supported by convergent evidence across multiple study designs, geographic contexts, and clinical settings:

- CPOE is associated with reductions in medication errors attributable to handwriting illegibility, transcription, and dose calculation of approximately 50%–54% (10, 21), with greater reductions in settings with high baseline error rates driven by these mechanisms (14, 15).
- Alert fatigue is a system-level phenomenon, not merely a failure of individual clinicians (27, 56), driven by alert volume, specificity, and quality of workflow integration. Override rates exceeding 90% for drug–drug interaction alerts are a robust finding across health systems and EHR platforms (16, 22).
- Human factors particularly nursing workload and interface design — are major determinants of real-world CDSS effectiveness (29, 44) and cannot be captured adequately by efficacy trials focused solely on prescribing-stage outcomes (51).
- NLP-based passive ADE surveillance substantially outperforms voluntary reporting for event detection within the training environment (41, 64), but performance degrades meaningfully when deployed in clinical contexts that differ from the training corpus (60).
- Technology-induced medication errors exist as a distinct, under-attributed category of iatrogenic harm (18, 42), although reliable incidence estimates are unavailable because of limitations in surveillance infrastructure (82).

### 5.2 Unresolved questions and genuine uncertainties

The following areas remain genuinely contested, methodologically limited, or unresolved:

- Whether adaptive, ML-based CDS alert generation reduces preventable ADEs at population level compared with standard rule-based systems in multisite, independently evaluated RCTs (21, 50); this remains the most important unresolved efficacy question in the field.
- The optimal regulatory model for AI-based clinical decision support (20, 30); no comparative evidence currently exists on the relative effectiveness of premarket review, post-market surveillance registries, or adaptive licensing in protecting patients from harm whilst enabling beneficial innovation (84).
- The magnitude and distribution of algorithmic inequity in deployed CDS systems (20, 37); although mechanisms are well described, reliable prevalence data from systematic equity audits are lacking (7).
- Whether the nursing-vigilance function can be reliably quantified and designed for (29, 79), or whether it is too context-sensitive and individual-dependent to serve as a systematic safety layer (80).
- The net safety impact of AI integration in medication safety over a 10–20-year horizon (19, 60), accounting for both improved detection and the risks of automation bias (51), skill atrophy, and technological dependence (24).

### 5.3 Limitations of this review

Several methodological limitations warrant explicit acknowledgement. First, the review protocol was not prospectively registered; whilst this is consistent with the narrative review design, it introduces the possibility of *post-hoc* thematic selection bias that a registered protocol would mitigate. Reviewer concern about the *a priori* status of the eight thematic domains is noted; these domains were derived from the review questions at the protocol design stage, but without prospective registration, this cannot be independently verified. The review is therefore framed explicitly as a structured narrative synthesis with interpretive elements, and thematic conclusions are not presented as though derived from a fully pre-specified protocol.

Second, the restriction to English-language publications introduces language bias, particularly regarding LMIC contexts. Evidence from non-English healthcare systems in East Asia, Latin America, and sub-Saharan Africa is likely underrepresented, as are studies indexed in LILACS and African Journals Online. All LMIC claims in this review are hedged accordingly.

Third, grey literature including regulatory guidance documents, health technology assessment reports, and government-commissioned reviews was not systematically searched, which may have resulted in underrepresentation of policy-relevant evidence, particularly for the governance and regulatory sections.

Fourth, the heterogeneity of included study designs limits the confidence with which aggregate conclusions can be drawn; the framework synthesis approach was selected precisely to accommodate this heterogeneity, but it cannot eliminate it, and the epistemological difference between quantitative efficacy evidence and qualitative implementation evidence is preserved in the synthesis.

Fifth, the CSIL framework represents a conceptual synthesis grounded in the reviewed evidence; it has not been empirically tested as a whole, and validation studies in diverse clinical settings are required before it can be applied as a practical implementation guide.

## 6 Research agenda

The following strategic research agenda synthesises the priority directions identified throughout this review, organised by methodological urgency and translational potential. To avoid redundancy with Section 3.5, Table 2, and Table 3, this section provides organisational framing rather than itemised repetition. The five research priorities identified in Section 3.5 constitute the core agenda. They are organised here by time horizon:

**Table 3:** Focused research agenda organised by priority domain.

Priority domain	Specific research questions (Review question)	Suggested approaches
Adaptive CDS architecture	Can CDS alerts be personalised to physician prescribing style and patient risk context without reducing safety? (RQ1, RQ3)	Reinforcement-learning models; A/B trials of tiered alert systems in EHR environments; prespecified equity subgroup analyses
Nursing-CDS interaction	What workload thresholds predict impaired CDS engagement, and how can CDS design buffer these effects? (RQ2)	Ecological momentary assessment; physiological workload monitoring + CDS interaction logging; prospective ICU and community settings
AI explainability in CDS	Do explainable AI outputs alter clinician trust, override behaviour, and ADE rates? (RQ3)	RCTs comparing black-box vs. XAI-enhanced CDS alerts; trust-calibration measurement; multisite designs
Technology-induced error taxonomy	What is the incidence and typology of EHR-mediated medication errors, and how do they interact with human factors? (RQ1, RQ2)	Prospective incident reporting with standardised e-iatrogenesis coding; national registry with EHR system identifiers
Equity-aware CDS	Do existing CDS systems generate differential alert rates or ADE outcomes by race, language, or socioeconomic status? (RQ3)	Intersectional audit studies; retraining CDS models on diverse datasets with fairness constraints; LMIC-focused studies
International regulatory harmonisation	Can adaptive licensing frameworks balance innovation speed with post-market surveillance for AI-CDS? (RQ3)	Comparative policy analysis; Delphi consensus across FDA, EMA, MHRA, TGA, and African regulatory bodies
CSIL empirical validation	Can the CSIL framework's five-node structure be validated as an organising model for medication-safety outcomes across diverse health systems?	Implementation science studies mapping node-specific interventions to outcome trajectories; mixed-methods evaluation in LMIC and high-income settings

Priority domains were identified from the evidence synthesis in Table 2. Research questions are framed to address core tensions for each domain and are linked to the review's research questions (RQ1–RQ3). Suggested approaches are illustrative and do not constitute a prescriptive research protocol. The CSIL empirical validation domain is added in this revision to address the need for prospective evaluation of the proposed framework. Regulatory agency abbreviations: FDA, US Food and Drug Administration; EMA, European Medicines Agency; MHRA, Medicines and Healthcare products Regulatory Agency (UK); TGA, Therapeutic Goods Administration (Australia).

Near term (0–3 years): evaluation infrastructure and measurement standardisation (18, 21). A standardised taxonomy for technology-induced medication errors (18, 42), adopted prospectively by a representative national sample of health systems, would transform surveillance capacity. Multisite RCTs comparing adaptive with rule-based CDS (21, 50), designed with ADE rate as the primary endpoint and pre-specified equity subgroup analyses, address the most important efficacy uncertainty in the field.

Medium term (3–7 years): translational implementation science. Comparative policy research examining regulatory models across jurisdictions with differing approaches to AI-CDS oversight (30). Systematic equity audits of commercially deployed systems in diverse health systems, specifically including LMICs and underserved populations (7, 37), constitute both a scientific and moral priority. LMIC-focused studies should consider expanding database searches to LILACS and African Journals Online, and to grey literature in local languages.

Long term (7–15 years): integration of CSIL components into learning-health-system infrastructure closed-loop systems in which ADE surveillance, AI model performance, and regulatory signals are analysed continuously and generate automated triggers for system refinement and regulatory escalation where safety thresholds are breached (24, 85). This ambition requires sustained collaboration among health informaticians, patient-safety researchers, AI developers, regulators, and patient representatives (36, 39).

## 7 Conclusion

The promise of digital health transformation for medication safety is real, but it has not yet been delivered reliably (2, 49). This review has shown that CDSS technology, despite decades of implementation and substantial global investment, produces highly variable real-world safety outcomes (14, 21), substantially attenuated by alert fatigue (16, 27), workflow mismatch (51), and interface-design failures that human-factors science has documented but system design has not yet remedied systematically (43, 44). The emergence of AI and ML introduces new dimensions of analytical capability (19, 60) but also new categories of risk: technology-induced error (18), algorithmic inequity (20, 37), automation bias (51), and regulatory lag (30).

The CSIL, proposed here as an integrative conceptual framework derived from the thematic synthesis, provides a relational architecture for understanding why these failures persist and what would be required to address them (36). It is a conceptual scaffold, not a validated implementation model, and its propositions require prospective empirical testing across diverse clinical and health system contexts. The framework's central diagnostic insight that the feedback loop between clinical outcomes and system learning (Node 5) is the least developed component of current AI-CDS deployments has direct implications for how health systems, AI developers, and regulators should prioritise investment.

Progress requires a systems-level response: evidence-based CDS design standards with regulatory force (30, 43); mandatory equity auditing of AI clinical decision tools (20, 37); prospective surveillance infrastructure for technology-induced errors (18, 42); and adaptive governance frameworks able to respond to emerging evidence more rapidly than current regulatory timelines allow (30). The research agenda presented here provides a pathway for generating the evidence base to support these responses. What is now required is institutional commitment across health systems, AI developers, regulators, and clinical professions to implement it (24, 36, 39).
