# Supplementary figures and images for: Integrating clinical decision support systems, nursing vigilance, and physician prescribing patterns to reduce preventable adverse drug events: a structured evidence-based narrative review on human-AI interface in medication safety
Source: Front Digit Health. 2026 Jul 7;8:1831150. doi: 10.3389/fdgth.2026.1831150 (PMC13386419; doi:10.3389/fdgth.2026.1831150)

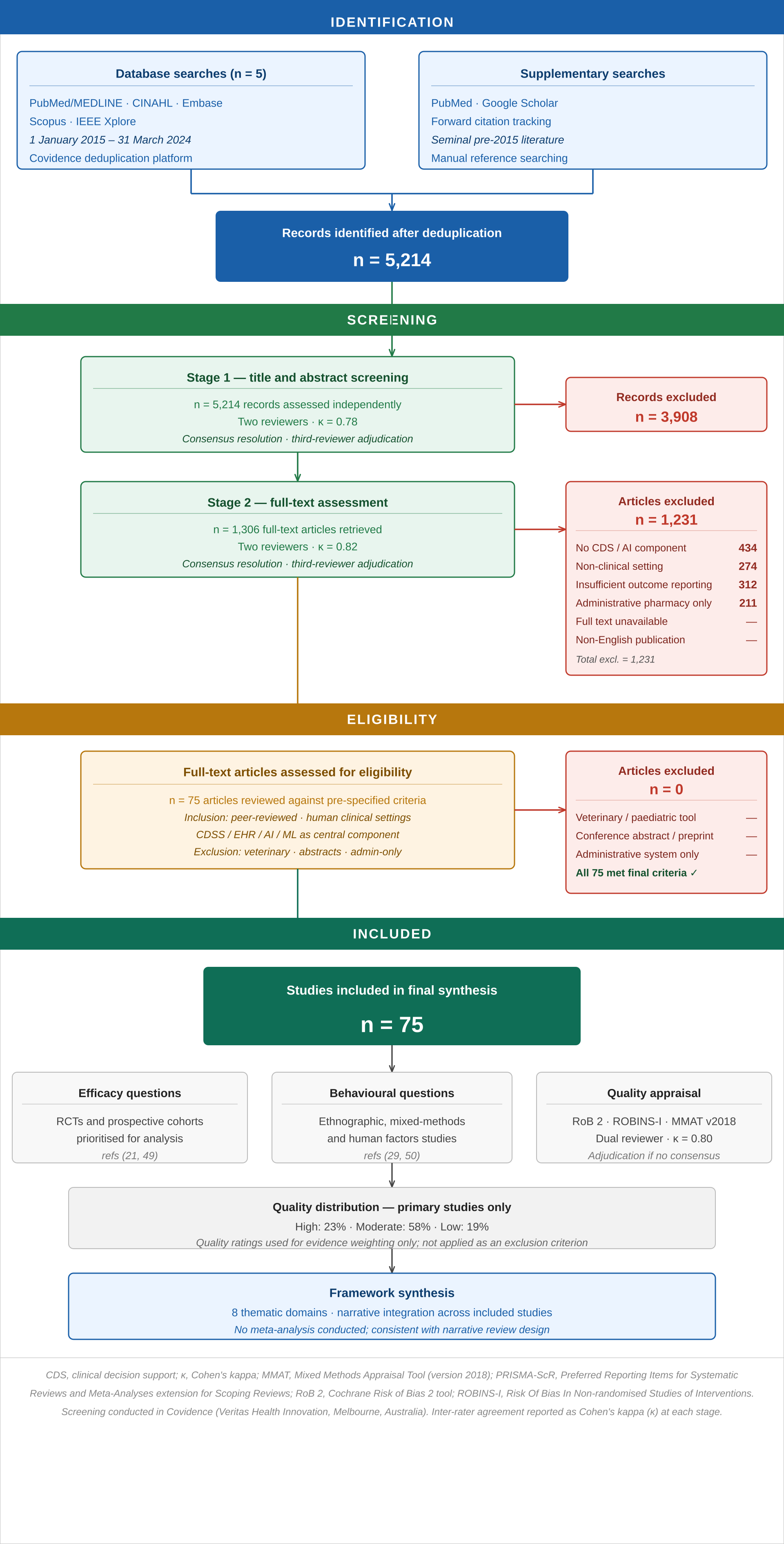

Supplement: Supplementary file 1 [file Image1.png]
