# Supplementary material for: Integrating clinical decision support systems, nursing vigilance, and physician prescribing patterns to reduce preventable adverse drug events: a structured evidence-based narrative review on human-AI interface in medication safety
Source: Front Digit Health. 2026 Jul 7;8:1831150. doi: 10.3389/fdgth.2026.1831150 (PMC13386419; doi:10.3389/fdgth.2026.1831150)
Supplement: Supplementary file 2 [file Table1.docx]

**Supplementary Table S1. Overview of the literature search strategy and study selection process**

This table provides a concise summary of the literature search strategy, databases searched, search period, key concepts, and study selection process undertaken for the structured evidence-based narrative review. Complete database-specific search strings and controlled vocabulary mappings are provided in Supplementary Table S2.

| **Component** | **Description** |
| --- | --- |
| **Review design** | Structured evidence-based narrative review conducted in accordance with the Scale for the Assessment of Narrative Review Articles (SANRA), with methodological reporting informed by the PRISMA-ScR guidance where applicable. |
| **Databases searched** | PubMed/MEDLINE, Embase, CINAHL, Scopus, and IEEE Xplore. |
| **Search period** | Literature published between 1 January 2015 and 31 March 2024, supplemented by targeted searches for seminal pre-2015 studies through PubMed and Google Scholar. |
| **Core search concepts** | Clinical decision support systems (CDSS); computerised physician order entry (CPOE); electronic health records (EHRs); medication safety; medication errors; adverse drug events (ADEs); artificial intelligence (AI); machine learning (ML); alert fatigue; nursing vigilance; prescribing patterns. |
| **Search strategy** | Database-specific Boolean search strings were developed using free-text keywords and controlled vocabulary (MeSH for PubMed/MEDLINE and Emtree for Embase where applicable). Manual reference list screening and forward citation tracking were also performed to identify additional eligible studies. |
| **Language restriction** | English-language publications only. |
| **Study types eligible** | Peer-reviewed primary studies, randomised controlled trials, observational studies, systematic reviews, meta-analyses, qualitative studies, mixed-methods studies, methodological studies, and narrative reviews relevant to medication safety and digital clinical decision support. |
| **Primary outcomes of interest** | Medication error rates; preventable adverse drug events; alert-response behaviour; clinician–AI interaction; CDSS effectiveness; AI-enabled medication safety; human factors; technology-induced errors; implementation outcomes. |

**Study selection summary**

| **Screening stage** | **Number of records** |
| --- | --- |
| Records identified after database searching and deduplication | **5,214** |
| Records excluded after title and abstract screening | **3,908** |
| Full-text articles assessed for eligibility | **1,306** |
| Full-text articles excluded | **1,231** |
| Studies included in the final narrative synthesis | **75** |

**Abbreviations:** ADE, adverse drug event; AI, artificial intelligence; CDSS, clinical decision support system; CPOE, computerised physician order entry; EHR, electronic health record; ML, machine learning; PRISMA-ScR, Preferred Reporting Items for Systematic Reviews and Meta-Analyses Extension for Scoping Reviews; SANRA, Scale for the Assessment of Narrative Review Articles.

**Table note:** This supplementary table summarises the overall search methodology and study selection process. The complete database-specific search strategies, Boolean operators, controlled vocabulary mappings, and supplementary search details are presented in Supplementary Table S2. The complete study-characteristics dataset is provided in Supplementary Table S3, while the full quality and risk-of-bias assessments are presented in Supplementary Table S4.
