# Supplementary material for: Integrating clinical decision support systems, nursing vigilance, and physician prescribing patterns to reduce preventable adverse drug events: a structured evidence-based narrative review on human-AI interface in medication safety
Source: Front Digit Health. 2026 Jul 7;8:1831150. doi: 10.3389/fdgth.2026.1831150 (PMC13386419; doi:10.3389/fdgth.2026.1831150)
Supplement: Supplementary file 3 [file Table2.docx]

# Supplementary Table S2

## Full Database-Specific Search Strings, Controlled Vocabulary Mappings, and Supplementary Searches

This table provides the complete search strategies applied across all five primary databases and the supplementary LILACS search. Primary searches were executed in March 2024. Database-specific controlled vocabulary terms (MeSH for PubMed/MEDLINE; Emtree for Embase; CINAHL Subject Headings; DeCS for LILACS) were mapped to free-text terms where supported. Secondary thematic searches targeted specific domains identified in the review protocol. The LILACS supplementary search was conducted in response to Reviewer 4's concern regarding LMIC evidence; no additional eligible studies were identified.

| **Database** | **Search Field Strategy** | **Full Search String** |
| --- | --- | --- |
| **PubMed/MEDLINE** | *MeSH + Free-text* | **Primary Boolean string:**  ("clinical decision support" OR "CDSS" OR "computerized physician order entry" OR "CPOE") AND ("medication error"[MeSH] OR "adverse drug event" OR "medication safety"[MeSH]) AND ("artificial intelligence"[MeSH] OR "machine learning"[MeSH] OR "alert fatigue" OR "nursing vigilance" OR "prescribing pattern")  **Secondary — AI/NLP for ADE detection:**  ("natural language processing"[MeSH] OR "text mining") AND ("adverse drug event" OR "adverse drug reaction"[MeSH]) AND ("electronic health records"[MeSH])  **Secondary — Technology-induced errors:**  ("medical errors"[MeSH] OR "medication errors"[MeSH]) AND ("electronic health records"[MeSH] OR "health information technology") AND ("iatrogenic disease"[MeSH] OR "technology" OR "interface" OR "usability")  **Secondary — EHR usability:**  ("electronic health records"[MeSH]) AND ("usability" OR "human factors" OR "workflow") AND ("medication safety" OR "patient safety"[MeSH])  **Secondary — Pharmacist-CDSS:**  ("pharmacists"[MeSH] OR "pharmaceutical services"[MeSH]) AND ("clinical decision support" OR "CDSS") AND ("medication errors"[MeSH])  **Secondary — Algorithmic bias:**  ("algorithms"[MeSH] OR "machine learning"[MeSH]) AND ("healthcare disparities"[MeSH] OR "bias" OR "racial groups"[MeSH]) AND ("clinical decision support")  **Date limits: 2015/01/01–2024/03/31**  **Language filter: English**  **Supplementary pre-2015 search: as above without date limit, limited to landmark/foundational works** |
| **CINAHL (via EBSCOhost)** | *CINAHL Subject Headings + Free-text* | **Primary Boolean string:**  ("clinical decision support" OR "CDSS" OR "computerized physician order entry" OR "CPOE") AND ("medication error" OR "adverse drug event" OR "medication safety") AND ("artificial intelligence" OR "machine learning" OR "alert fatigue" OR "nursing vigilance" OR "prescribing pattern")  CINAHL Subject Headings applied where available:  MH "Decision Support Systems, Clinical+" AND MH "Medication Errors+" AND (MH "Artificial Intelligence" OR MH "Machine Learning" OR TI "alert fatigue" OR AB "alert fatigue")  **Secondary nursing-focused string:**  (MH "Nurses+" OR MH "Nursing Care+") AND MH "Medication Administration+" AND ("clinical decision support" OR "CDSS" OR "alert" OR "vigilance")  **Date limits: 2015/01/01–2024/03/31**  **Language filter: English** |
| **Embase** | *Emtree + Free-text* | **Primary Boolean string (Emtree controlled vocabulary mapped):**  ('clinical decision support system'/exp OR 'CDSS' OR 'computerized physician order entry'/exp OR 'CPOE') AND ('medication error'/exp OR 'adverse drug reaction'/exp OR 'medication safety') AND ('artificial intelligence'/exp OR 'machine learning'/exp OR 'alert fatigue' OR 'nursing vigilance' OR 'prescribing behavior'/exp)  **Secondary — AI/ML pharmacovigilance:**  ('pharmacovigilance'/exp OR 'drug safety'/exp) AND ('machine learning'/exp OR 'natural language processing'/exp OR 'deep learning'/exp)  **Secondary — Technology-induced error:**  ('medical error'/exp OR 'medication error'/exp) AND ('health information system'/exp OR 'electronic health record'/exp) AND ('human error'/exp OR 'usability' OR 'interface')  **Date limits: 2015-2024**  **Language filter: English**  **Publication type filter: article, review, letter (excluded: editorial, conference abstract)** |
| **Scopus** | *Title/Abstract/Keywords* | **Primary Boolean string:**  TITLE-ABS-KEY(("clinical decision support" OR "CDSS" OR "computerized physician order entry" OR "CPOE") AND ("medication error" OR "adverse drug event" OR "medication safety") AND ("artificial intelligence" OR "machine learning" OR "alert fatigue" OR "nursing vigilance"))  **Secondary — Equity/bias:**  TITLE-ABS-KEY(("algorithmic bias" OR "racial bias" OR "health disparities") AND ("clinical decision support" OR "artificial intelligence") AND ("medication" OR "drug"))  **Secondary — Regulatory/governance:**  TITLE-ABS-KEY(("regulatory" OR "governance" OR "adaptive licensing") AND ("clinical decision support" OR "artificial intelligence") AND ("medication safety" OR "patient safety"))  **Date limits: 2015–2024**  **Language filter: English**  **Document type: article, review** |
| **IEEE Xplore** | *All Metadata* | **Primary Boolean string:**  ("clinical decision support" OR "CDSS" OR "physician order entry") AND ("medication error" OR "adverse drug event" OR "medication safety") AND ("artificial intelligence" OR "machine learning" OR "alert fatigue" OR "deep learning" OR "natural language processing")  **Secondary — AI/ML systems:**  ("electronic health record" OR "EHR") AND ("machine learning" OR "deep learning" OR "neural network") AND ("medication" OR "drug" OR "adverse event")  ***Note: IEEE Xplore searched for computational/informatics studies involving AI-CDSS integration; health informatics conference proceedings excluded at full-text screening per eligibility criteria.***  **Date limits: 2015–2024**  **Language filter: English** |
| **LILACS (Supplementary search, post-review)** | *DeCS Subject Headings + Free-text (Spanish)* | **Supplementary search conducted in response to Reviewer 4 concern regarding LMIC evidence.**  **Search string (Spanish):**  ("sistemas de apoyo a decisiones clínicas" OR "CDSS" OR "entrada computarizada de órdenes médicas") AND ("errores de medicación" OR "eventos adversos por medicamentos" OR "seguridad de medicamentos") AND ("inteligencia artificial" OR "aprendizaje automático" OR "fatiga de alertas")  **DeCS terms applied: sistemas de apoyo a decisiones clínicas; errores de medicación; inteligencia artificial; aprendizaje automático; registros electrónicos de salud.**  **Date limits: 2015–2024**  **Language filter: Spanish and Portuguese (to maximise LMIC coverage)**  ***Result: 0 records identified meeting full eligibility criteria (human clinical setting, CDS/AI/EHR component, medication safety outcome, full text available). No studies added from LILACS search.***  ***Note: African Journals Online (AJOL) was assessed; the available indexed content in this domain did not yield eligible studies meeting full-text criteria within the review's scope.*** |

*Abbreviations: CDS, clinical decision support; CDSS, clinical decision support system; CPOE, computerised physician order entry; DeCS, Descriptores en Ciencias de la Salud; EHR, electronic health record; LMIC, low- and middle-income country; MeSH, Medical Subject Headings.*

*Note: All searches restricted to peer-reviewed publications in English (and Spanish/Portuguese for LILACS). Conference abstracts, preprints, and dissertations were excluded at Stage 1 screening. Complete search execution dates: Primary databases March 2024; LILACS supplementary April 2024 (post-review). Full search logs are available from the corresponding author on request.*
