# Supplementary material for: Integrating clinical decision support systems, nursing vigilance, and physician prescribing patterns to reduce preventable adverse drug events: a structured evidence-based narrative review on human-AI interface in medication safety
Source: Front Digit Health. 2026 Jul 7;8:1831150. doi: 10.3389/fdgth.2026.1831150 (PMC13386419; doi:10.3389/fdgth.2026.1831150)
Supplement: Supplementary file 4 [file Table3.docx]

# Supplementary Table S3

## Study Characteristics Table All 75 Included Studies

This table presents the study characteristics for all 75 studies included in the final synthesis. Studies are sequenced by thematic domain, consistent with the eight a priori domains applied in the framework synthesis approach. Quality ratings were assigned using RoB 2 (RCTs), ROBINS-I (non-randomised intervention studies), or MMAT version 2018 (qualitative/mixed-methods studies), as described in Methods Section 2.7. Quality ratings informed evidence weighting during synthesis and were not used as a basis for exclusion. Inter-rater agreement for quality appraisal: κ = 0.80.

| **#** | **Authors (Year)** | **Study Design** | **Setting / Country** | **Population** | **CDS / AI System Description** | **Primary Outcome / Finding** | **Quality Rating** | **Thematic Domain** |
| --- | --- | --- | --- | --- | --- | --- | --- | --- |
| **1** | **Kaushal et al. (2003)** | Systematic review | US academic medical centres | Inpatient settings | CPOE + CDSS (rule-based) | Medication error reduction 55–83% following CPOE implementation | **High** | *CPOE Efficacy* |
| **2** | **Garg et al. (2005)** | Systematic review (100 trials) | Multisite; USA/Europe | Clinician users | Computerised CDS (multiple types) | CDS improved practitioner performance in 62% of studies | **High** | *CPOE Efficacy* |
| **3** | **Kawamoto et al. (2005)** | Systematic review | Global | Clinicians | CDSS (actionable recommendations) | 4 features predicted CDS success: integration, automatic provision, recommendations, decision-point triggering | **High** | *CPOE Efficacy* |
| **4** | **Hunt et al. (1998)** | Systematic review | USA | Physicians | Computer-based CDS | CDS improved physician performance in 66% of studies; patient outcomes improved in 43% | **High** | *CPOE Efficacy* |
| **5** | **Nuckols et al. (2014)** | Systematic review & meta-analysis (41 studies) | US hospitals | Inpatients | CPOE/CDS | CPOE associated with ~54% reduction in transcription/illegibility errors | **High** | *CPOE Efficacy* |
| **6** | **Nuckols et al. (2015)** | Economic modelling | USA acute care | Inpatients | CPOE implementation | Estimated substantial cost savings from CPOE adoption at scale | **Moderate** | *CPOE Efficacy* |
| **7** | **Metzger et al. (2010)** | Multi-site observational | US hospitals | Inpatient medication orders | CPOE | Mixed safety performance; alert fatigue and workflow issues identified | **Moderate** | *CPOE Efficacy* |
| **8** | **Eslami et al. (2008)** | Systematic review | USA/Europe | Inpatients | Computerised physician medication order entry | Beneficial impact on medication safety; variable effect sizes | **High** | *CPOE Efficacy* |
| **9** | **Ye & Bronstein (2025)** | RCT (shared CDS) | USA, inpatient | Clinicians, inpatients | Shared CDS for ADE reduction | Reductions in preventable ADEs using shared CDS framework | **Moderate** | *CPOE Efficacy* |
| **10** | **Shah et al. (2021)** | Multicentre inpatient study | US academic hospitals | Inpatient renal patients | Context-tailored renal CDS (commercial EHR) | Reduction in clinically inappropriate alert overrides vs standard alerting | **Moderate** | *CPOE Efficacy* |
| **11** | **Slight et al. (2013)** | Mixed-methods | UK NHS primary care | Prescribers/nurses | Drug–drug interaction alerts (EHR-integrated) | Override rate >90%; dose–response between alert burden and override behaviour | **Moderate** | *Alert Fatigue* |
| **12** | **van der Sijs et al. (2006)** | Observational | Netherlands; multiple hospitals | Prescribers | CPOE drug safety alerts | 87% of DDI alerts overridden; alert fatigue documented | **Moderate** | *Alert Fatigue* |
| **13** | **Ancker et al. (2017)** | Mixed-methods | USA, CDS users | Clinicians | EHR-integrated CDS (alert system) | Workload, complexity, and repeated alerts associated with alert fatigue via dual-process mechanism | **Moderate** | *Alert Fatigue* |
| **14** | **Co et al. (2020)** | Retrospective analysis | USA, large health system | Prescribers | CPOE alert system | Tradeoff between safety and alert fatigue quantified; override patterns analysed | **Moderate** | *Alert Fatigue* |
| **15** | **Phansalkar et al. (2012)** | Consensus/expert panel | USA | Clinical experts | DDI alert prioritisation | High-priority DDI alert framework developed to reduce low-value alerts | **Moderate** | *Alert Fatigue* |
| **16** | **Seidling et al. (2011)** | Predictive modelling | Multi-institutional | Prescribers | CDS alert system | Factors predicting alert acceptance identified; actionability key determinant | **Moderate** | *Alert Fatigue* |
| **17** | **Clarke et al. (2025)** | Observational | USA clinical informatics | Clinicians | Highly targeted alert system | Alert burden characterised; specificity-fatigue tradeoff demonstrated | **Moderate** | *Alert Fatigue* |
| **18** | **Embi & Leonard (2012)** | Longitudinal observational | USA, academic hospital | Clinicians (clinical trial alerts) | EHR clinical trial alerts | Alert fatigue increased over time; habituation effect documented | **Moderate** | *Alert Fatigue* |
| **19** | **Payne et al. (2002)** | Descriptive | USA, academic hospital | Physicians | CPOE order-check system | High override rates; characteristics of commonly overridden checks described | **Low** | *Alert Fatigue* |
| **20** | **Nanji et al. (2014)** | Observational | USA, ambulatory | Outpatient prescribers | CDS medication alerts | High override rates in outpatient CDS; contextual factors identified | **Moderate** | *Alert Fatigue* |
| **21** | **Carayon et al. (2014)** | Human factors review | USA, 12 ICU hospitals | ICU nurses | ICU nursing workflow–CDS interaction | Nurses function as safety buffer; workload mediates CDS response accuracy | **Moderate** | *Nursing Vigilance* |
| **22** | **Smeulers et al. (2014)** | Cochrane systematic review | Hospitalised patients (global) | Nurses | Nursing handover practices | Handover style affects continuity of care; systemic safety framing (indirect vigilance context) | **High** | *Nursing Vigilance* |
| **23** | **Holden et al. (2013)** | Usability study, 14 hospitals | USA, multi-hospital | Nurses + CDS interaction | SEIPS 2.0 / CDS interface | Alert screen position altered override rates by ~22%; human factors analysis | **Moderate** | *Nursing Vigilance* |
| **24** | **Rayo & Moffatt-Bruce (2015)** | Evidence review | USA, alarm management | Clinicians | Alarm and alert system management | Direct measurement of alert informativeness improves alarm response; vigilance framing | **Moderate** | *Nursing Vigilance* |
| **25** | **Chui & Mott (2012)** | Survey/observational | USA, community pharmacy | Pharmacists | Community pharmacy workload | Workload inversely associated with task performance; human factors perspective | **Moderate** | *Nursing Vigilance* |
| **26** | **Prgomet et al. (2017)** | Systematic review | ICUs, global | ICU patients, clinicians | Commercial CPOE + CDS | CPOE/CDS impact on medication errors, LOS, mortality in ICUs | **Moderate** | *Nursing Vigilance* |
| **27** | **Helmons et al. (2009)** | Pre-post observational | USA hospital | Nurses, medication administration | Bar-code medication administration (BCMA) | BCMA reduced medication administration errors; nursing safety layer evidenced | **Moderate** | *Nursing Vigilance* |
| **28** | **Poon et al. (2010)** | Cluster RCT | USA academic hospital | Nurses, inpatient medication admin | Bar-code technology | Bar-code technology reduced medication administration errors | **High** | *Nursing Vigilance* |
| **29** | **Gurses et al. (2010)** | Multi-method study | USA, ICU | Clinicians | Evidence-based guidelines compliance | Factors affecting guideline compliance identified; workload key barrier | **Moderate** | *Nursing Vigilance* |
| **30** | **Sujan et al. (2016)** | Sociotechnical analysis | UK/Europe | Healthcare system actors | Sociotechnical systems (complex adaptive) | Trade-offs and residual risks in sociotechnical safety systems characterised | **Moderate** | *Nursing Vigilance* |
| **31** | **Magrabi et al. (2012)** | Incident analysis | USA (FDA safety reports) | Healthcare system, HIT users | EHR/HIT systems (multiple) | Classification of HIT safety problems; EHR-related incidents systematically under-attributed | **Moderate** | *Technology-Induced Error* |
| **32** | **Samaranayake et al. (2012)** | Retrospective incident analysis (5 years) | Hong Kong tertiary hospital | Inpatients, medication safety | EHR-integrated medication system | Technology-related medication errors identified; drop-down errors, auto-population mechanisms | **Moderate** | *Technology-Induced Error* |
| **33** | **Schiff et al. (2015)** | Error analysis / vulnerability testing | USA health systems | Clinicians, CPOE users | CPOE (commercial systems) | CPOE-related medication errors identified; regulatory responses frequently delayed | **Moderate** | *Technology-Induced Error* |
| **34** | **Brown et al. (2017)** | Systematic review | Global, CPOE settings | Clinicians, inpatients | Computerised provider order entry | Typology of prescribing errors from CPOE use described; interface failures prominent | **High** | *Technology-Induced Error* |
| **35** | **Ash et al. (2006)** | Ethnographic, 18 sites | USA, EHR-integrated | Clinicians, health system | EHR-integrated CDS | Workflow mismatch at 14/18 sites; CPOE implementation associated with power shifts | **Moderate** | *Technology-Induced Error* |
| **36** | **Amato et al. (2017)** | Incident analysis | USA, patient safety reports | Healthcare system | CPOE (multiple vendors) | 2,522 CPOE-related medication errors analysed; interface design implicated | **Moderate** | *Technology-Induced Error* |
| **37** | **Ash et al. (2004)** | Qualitative analysis | USA, multiple settings | Clinicians, health systems | Information technology in healthcare | Unintended consequences of health IT identified; systematic framework proposed | **Moderate** | *Technology-Induced Error* |
| **38** | **Graber et al. (2015)** | Medical malpractice analysis | USA | Health systems, legal records | EHR systems (multiple) | EHR-related events in malpractice claims characterised; under-reporting documented | **Low** | *Technology-Induced Error* |
| **39** | **Ahmed et al. (2011)** | Controlled experiment | USA, ICU setting | ICU providers | Two different EHR user interfaces | Interface differences affected task load, cognitive errors, and performance | **Moderate** | *Technology-Induced Error* |
| **40** | **Middleton et al. (2013)** | Expert consensus / policy | USA | Clinicians, health informatics | EHR usability (general) | EHR usability framework for patient safety and quality improvement described | **Moderate** | *Technology-Induced Error* |
| **41** | **Harpaz et al. (2014)** | Narrative review | Global | Pharmacovigilance systems | Text mining / NLP for ADE detection | State of the art in text mining for ADEs; promise and challenges described | **Moderate** | *AI/ML Applications* |
| **42** | **Murff et al. (2011)** | NLP validation study | USA, academic hospital (surgical) | EHR records, surgical patients | NLP-based automated complication detection | NLP identified postoperative complications at rates exceeding manual chart review | **Moderate** | *AI/ML Applications* |
| **43** | **Jeong et al. (2018)** | ML model development | South Korea, academic hospital | Laboratory event data | Ensemble ML model for ADE signal detection | ML combining multiple algorithms improved ADE signal detection from lab data | **Moderate** | *AI/ML Applications* |
| **44** | **Nistal-Nuno (2022)** | Algorithm development | Online pharmacy (Spain) | Pharmacy users | Adaptive user interface for medication recommendation | Adaptive recommendation system improved appropriateness of drug selection | **Low** | *AI/ML Applications* |
| **45** | **Khairat et al. (2018)** | Critical analysis | USA | Physicians, CDS users | CDSS (general) | Barriers to physician adoption of CDS identified; trust and workflow integration key | **Moderate** | *AI/ML Applications* |
| **46** | **Bellini et al. (2022)** | Systematic review | Global, perioperative | Anaesthesiology, perioperative | ML in perioperative medicine | ML applications in perioperative medicine reviewed; evidence emerging-moderate | **Moderate** | *AI/ML Applications* |
| **47** | **Alghamdi (2025)** | Narrative review | Global | Drug discovery, neurological | AI-driven drug discovery | AI role in drug discovery against neurological disorders; broader AI framework | **Low** | *AI/ML Applications* |
| **48** | **Martins et al. (2018)** | Comparative study | Brazil, ICU | ICU patients | Trigger tools vs chart review for ADE detection | Trigger tools equivalent to non-targeted chart review for ADE detection | **Moderate** | *AI/ML Applications* |
| **49** | **Evans et al. (2001)** | Methodological | Global pharmacovigilance | Spontaneous ADE reports | Proportional reporting ratios (PRRs) | PRR method for pharmacovigilance signal generation validated | **Moderate** | *AI/ML Applications* |
| **50** | **Topol (2019)** | Narrative review | Global | Clinicians, healthcare systems | AI in clinical medicine (multiple) | AI should augment rather than replace clinical cognition; high-performance medicine framework | **Moderate** | *AI/ML Applications* |
| **51** | **Wright & Sittig (2008)** | Framework analysis | Global, CDS architecture | CDS system developers, clinicians | CDS architectures (multiple) | Alert specificity core design challenge; framework for evaluating CDS architectures | **Moderate** | *Human Factors* |
| **52** | **Ash et al. (2006b)** | Multi-site ethnographic | USA, 18 systems | Clinicians, EHR users | EHR-integrated CDS | Workflow mismatch; unintended consequences of CPOE characterised | **Moderate** | *Human Factors* |
| **53** | **McCoy et al. (2014)** | Review and proposal | USA | Clinicians, CDS users | CDS alert appropriateness | Framework for improving CDS alert appropriateness proposed | **Moderate** | *Human Factors* |
| **54** | **Lipsitz (2012)** | Conceptual analysis | USA | Healthcare systems | Complex adaptive systems | Healthcare framed as complex system; non-linear dynamics and emergent behaviour | **Moderate** | *Human Factors* |
| **55** | **Dy & Purnell (2012)** | Conceptual review | USA, healthcare | Patients, clinicians | Complex and shared decision-making | Key quality concepts for shared decision-making in complex care identified | **Moderate** | *Human Factors* |
| **56** | **Steyerberg & Vergouwe (2014)** | Methodological (clinical prediction) | Global | Clinical prediction model developers | Statistical modelling methods | 7-step framework for prediction model development and ABCD validation standard | **High** | *Human Factors* |
| **57** | **Holstiege et al. (2015)** | Systematic review | Global, primary care | Primary care providers | Computer-aided CDS for antibiotic prescribing | CDS improved antibiotic prescribing appropriateness in primary care | **Moderate** | *Human Factors* |
| **58** | **Shojania et al. (2010)** | Systematic review | Global | Physicians | Point-of-care computer reminders | Modest improvement in physician compliance; reminder design critical | **High** | *Human Factors* |
| **59** | **Classen et al. (2011)** | Expert consensus | USA | Clinicians, informaticists | Drug–drug interaction lists (EHR-integrated) | Critical DDI list for CPOE systems developed; risk-stratified | **Moderate** | *Human Factors* |
| **60** | **Phansalkar et al. (2012b)** | Expert consensus | USA | Clinicians, pharmacists | High-priority DDI alerts (EHR) | High-priority DDI framework to guide alert curation for EHR systems | **Moderate** | *Human Factors* |
| **61** | **Obermeyer et al. (2019)** | Retrospective analysis | USA, commercial algorithm | Black and White patients | Widely deployed care-management algorithm | Racial bias: systematic under-identification of need in Black patients | **High** | *Algorithmic Equity* |
| **62** | **Roberti et al. (2024)** | Cross-sectional survey (4 countries) | Latin America | Adult patients, health system users | Health system quality and coverage | Substantial inequalities in health system quality across 4 countries; LMIC vulnerability evidenced | **Moderate** | *Algorithmic Equity* |
| **63** | **Kiguba et al. (2023)** | Narrative review | Sub-Saharan Africa / LMICs | LMIC pharmacovigilance systems | Pharmacovigilance systems | Constraints on pharmacovigilance in LMICs; ADE burden likely underestimated | **Moderate** | *Algorithmic Equity* |
| **64** | **Campanella et al. (2016)** | Systematic review & meta-analysis | Europe, global | Inpatients, health system | Electronic health records (general) | EHR positive impact on healthcare quality; heterogeneity in effect sizes | **High** | *Algorithmic Equity* |
| **65** | **Neves et al. (2020)** | Systematic review & meta-analysis | Global | Patients with EHR access | Patient-facing EHR access | EHR patient access associated with improved quality and safety | **High** | *Algorithmic Equity* |
| **66** | **Dullabh et al. (2022)** | Landscape analysis | USA | CDS developers, policymakers | Patient-centred CDS technical landscape | Progress, gaps, and challenges in patient-centred CDS landscape identified | **Moderate** | *Regulatory/Governance* |
| **67** | **Sittig & Singh (2010)** | Conceptual framework | USA/global | Health informaticians | Sociotechnical model (8 dimensions) | New sociotechnical model for studying HIT in complex adaptive systems | **Moderate** | *Regulatory/Governance* |
| **68** | **Coiera (2015)** | Textbook/reference | Global | Health informaticians | Health informatics (comprehensive) | Guide to health informatics; foundational reference for health IT and CDS | **High** | *Regulatory/Governance* |
| **69** | **Baethge et al. (2019)** | Methodological | Global | Review authors | Narrative review methodology | SANRA framework for quality assessment of narrative reviews validated | **Moderate** | *Regulatory/Governance* |
| **70** | **Wang et al. (2017)** | Methodological | Global | Pharmacoepidemiology researchers | Healthcare database studies | Reporting standards for pharmacoepidemiology and database studies | **High** | *Regulatory/Governance* |
| **71** | **Bastoni et al. (2021)** | Umbrella review | Europe, global | eHealth users, informal carers | eHealth technologies (dementia care) | Implementation barriers and facilitators for eHealth; governance and adoption factors | **Moderate** | *Regulatory/Governance* |
| **72** | **Walsh et al. (2017)** | Systematic review | Global | Healthcare systems | Medication error (economic) | Economic impact of medication error; substantial costs across settings | **High** | *Regulatory/Governance* |
| **73** | **Bayoumi et al. (2014)** | Observational, population-based | Canada, older adults | Older adults, emergency settings | Medication-related ED visits | Medication-related ED visits and hospitalisations in older adults characterised | **Moderate** | *Regulatory/Governance* |
| **74** | **Gandhi et al. (2003)** | Prospective cohort | USA, ambulatory | Ambulatory patients, prescribers | Ambulatory medication safety | ADEs common in ambulatory care; preventable in substantial proportion | **High** | *Regulatory/Governance* |
| **75** | **Agrawal (2009)** | Narrative review | Global | Clinicians, policymakers | IT systems for medication error prevention | IT-based approaches to medication error prevention reviewed | **Moderate** | *Regulatory/Governance* |

*Quality ratings: High = low risk of bias / high methodological quality; Moderate = some concerns / methodological limitations present; Low = high risk of bias or insufficient reporting. Quality grading is design-appropriate and was not applied uniformly across study types.*

*Abbreviations: AI, artificial intelligence; CDS, clinical decision support; CDSS, clinical decision support system; CPOE, computerised physician order entry; DDI, drug–drug interaction; EHR, electronic health record; ICU, intensive care unit; LMIC, low- and middle-income country; ML, machine learning; NLP, natural language processing; RCT, randomised controlled trial.*
