# Supplementary material for: Integrating clinical decision support systems, nursing vigilance, and physician prescribing patterns to reduce preventable adverse drug events: a structured evidence-based narrative review on human-AI interface in medication safety
Source: Front Digit Health. 2026 Jul 7;8:1831150. doi: 10.3389/fdgth.2026.1831150 (PMC13386419; doi:10.3389/fdgth.2026.1831150)
Supplement: Supplementary file 5 [file Table4.docx]

**Supplementary Table S4**

**Full Quality and Risk of Bias Appraisal Records All Appraised Studies**

This table presents the full quality appraisal records for studies included in the synthesis, organised by appraisal instrument. Quality was appraised using: the Cochrane Risk of Bias 2 (RoB 2) tool for RCTs and systematic reviews of RCTs; the Risk Of Bias In Non-randomised Studies of Interventions (ROBINS-I) tool for non-randomised intervention studies and observational designs; and the Mixed Methods Appraisal Tool (MMAT, version 2018) for qualitative, mixed-methods, narrative reviews, and modelling studies. Appraisal was performed independently by two reviewers; inter-rater agreement: κ = 0.80. Disagreements were resolved by consensus or third-reviewer adjudication. Quality ratings informed evidence weighting during synthesis and were not used as a basis for exclusion.

Domain labels are generalised across appraisal instruments: D1 (Confounding/Selection Bias), D2 (Measurement/Outcome Validity), D3 (Reporting Completeness), D4 (Generalisability/External Validity). For AI-specific studies, four additional criteria were applied (training-validation split; external validation; model performance metrics; explainability transparency), and findings are noted in the appraisal notes column. For systematic reviews and meta-analyses appraised with RoB 2, domain judgements reflect the aggregate risk across included primary studies.

| **#** | **Authors (Year)** | **Appraisal Tool** | **D1: Confounding / Selection Bias** | **D2: Measurement / Outcome Validity** | **D3: Reporting Completeness** | **D4: Generalisability / External Validity** | **Overall Rating** | **Appraisal Notes** |
| --- | --- | --- | --- | --- | --- | --- | --- | --- |
| **1** | **Kaushal et al. (2003)** | RoB 2 | **L** | **L** | **L** | **M** | **High** | Systematic review; broad scope; some heterogeneity in primary study design |
| **2** | **Garg et al. (2005)** | RoB 2 | **L** | **L** | **L** | **M** | **High** | 100 RCTs/CCTs; rigorous synthesis; variation in CDS types limits pooling |
| **3** | **Kawamoto et al. (2005)** | RoB 2 | **L** | **L** | **L** | **M** | **High** | Systematic review; 4 success features; limited to prescribing outcomes |
| **4** | **Hunt et al. (1998)** | RoB 2 | **L** | **L** | **L** | **M** | **High** | Foundational systematic review; older evidence base; design heterogeneity |
| **5** | **Nuckols et al. (2014)** | RoB 2 | **L** | **L** | **L** | **M** | **High** | Meta-analysis; rigorous; heterogeneity in error definitions across studies |
| **28** | **Poon et al. (2010)** | RoB 2 | **L** | **L** | **L** | **M** | **High** | Cluster RCT; BCMA; strong design; single academic site limits generalisability |
| **64** | **Campanella et al. (2016)** | RoB 2 | **L** | **M** | **L** | **M** | **High** | Systematic review + meta-analysis; heterogeneity in EHR definitions |
| **65** | **Neves et al. (2020)** | RoB 2 | **L** | **L** | **L** | **M** | **High** | Systematic review + meta-analysis; patient-facing EHR; broad outcomes |
| **70** | **Wang et al. (2017)** | RoB 2 | **L** | **L** | **L** | **L** | **High** | Methodological reporting standard; international authorship; widely adopted |
| **74** | **Gandhi et al. (2003)** | RoB 2 | **L** | **L** | **L** | **M** | **High** | Prospective cohort; ambulatory setting; self-report of some outcomes |
| **72** | **Walsh et al. (2017)** | RoB 2 | **L** | **L** | **L** | **M** | **High** | Systematic review; economic focus; heterogeneity in cost measurement |
| **7** | **Metzger et al. (2010)** | ROBINS-I | **M** | **M** | **M** | **M** | **Moderate** | Multi-site pre-post; variation in CPOE systems; no randomisation; secular trends possible |
| **10** | **Shah et al. (2021)** | ROBINS-I | **M** | **L** | **M** | **M** | **Moderate** | Multicentre; pre-post; US academic hospitals only; external validity uncertain |
| **16** | **Seidling et al. (2011)** | ROBINS-I | **M** | **M** | **L** | **M** | **Moderate** | Predictive modelling; single-centre basis for derivation; limited validation |
| **22** | **Smeulers et al. (2014)** | ROBINS-I | **L** | **L** | **L** | **M** | **High** | Cochrane review of handover studies; high internal quality; indirect relevance to CDSS |
| **27** | **Helmons et al. (2009)** | ROBINS-I | **M** | **M** | **M** | **M** | **Moderate** | Pre-post design; BCMA; Hawthorne effect possible; single US hospital |
| **33** | **Schiff et al. (2015)** | ROBINS-I | **M** | **M** | **M** | **M** | **Moderate** | Vulnerability testing + incident analysis; US data only; under-reporting acknowledged |
| **36** | **Amato et al. (2017)** | ROBINS-I | **M** | **M** | **M** | **M** | **Moderate** | Large incident report dataset; attribution bias; US voluntary reporting limitations |
| **43** | **Jeong et al. (2018)** | ROBINS-I | **M** | **M** | **M** | **H** | **Moderate** | Single Korean hospital; external validation limited; performance metrics partial |
| **62** | **Roberti et al. (2024)** | ROBINS-I | **M** | **M** | **L** | **M** | **Moderate** | Cross-sectional; 4 Latin American countries; self-report/administrative data mix |
| **66** | **Dullabh et al. (2022)** | ROBINS-I | **M** | **M** | **M** | **M** | **Moderate** | USA-focused landscape analysis; expert consensus elements; limited primary data |
| **6** | **Nuckols et al. (2015)** | MMAT | **M** | **M** | **M** | **M** | **Moderate** | Economic modelling; model assumptions not fully reported; US hospital context |
| **8** | **Eslami et al. (2008)** | MMAT | **M** | **L** | **M** | **M** | **Moderate** | Systematic review; older literature; definition heterogeneity |
| **9** | **Ye & Bronstein (2025)** | MMAT | **M** | **M** | **M** | **M** | **Moderate** | RCT; shared CDS intervention; single system; recent but limited replication |
| **11** | **Slight et al. (2013)** | MMAT | **M** | **M** | **M** | **M** | **Moderate** | Mixed-methods; UK NHS; self-report vigilance; cross-sectional elements |
| **12** | **van der Sijs et al. (2006)** | MMAT | **M** | **M** | **L** | **M** | **Moderate** | Observational; Netherlands; single hospital system; no comparator group |
| **13** | **Ancker et al. (2017)** | MMAT | **M** | **M** | **M** | **M** | **Moderate** | Mixed-methods; USA CDS; dual-process model theoretically derived; limited empirical testing |
| **14** | **Co et al. (2020)** | MMAT | **M** | **M** | **M** | **M** | **Moderate** | Retrospective; large US health system; observational causal attribution limited |
| **15** | **Phansalkar et al. (2012)** | MMAT | **M** | **M** | **M** | **M** | **Moderate** | Expert consensus; limited empirical testing of proposed prioritisation scheme |
| **17** | **Clarke et al. (2025)** | MMAT | **M** | **M** | **M** | **M** | **Moderate** | Single-site observational; recent; limited replication; alert characterisation solid |
| **18** | **Embi & Leonard (2012)** | MMAT | **M** | **M** | **M** | **M** | **Moderate** | Longitudinal; alert fatigue characterised; single institution; clinical trial alerts only |
| **19** | **Payne et al. (2002)** | MMAT | **M** | **H** | **H** | **H** | **Low** | Descriptive; older evidence; single-site CPOE; limited outcome data |
| **20** | **Nanji et al. (2014)** | MMAT | **M** | **M** | **M** | **M** | **Moderate** | Observational; ambulatory; override rates; no outcome linkage |
| **21** | **Carayon et al. (2014)** | MMAT | **M** | **M** | **M** | **M** | **Moderate** | Human factors review; ICU setting; cross-sectional elements; US-centred |
| **23** | **Holden et al. (2013)** | MMAT | **M** | **M** | **M** | **M** | **Moderate** | Usability study; 14 hospitals; SEIPS 2.0; effect size specific to study conditions |
| **24** | **Rayo & Moffatt-Bruce (2015)** | MMAT | **M** | **M** | **M** | **M** | **Moderate** | Evidence review; alarm management; no primary data collection |
| **25** | **Chui & Mott (2012)** | MMAT | **M** | **M** | **M** | **M** | **Moderate** | Survey; community pharmacy; subjective workload measures; USA only |
| **26** | **Prgomet et al. (2017)** | MMAT | **M** | **M** | **M** | **M** | **Moderate** | Systematic review; ICU; heterogeneous CPOE types; publication bias possible |
| **29** | **Gurses et al. (2010)** | MMAT | **M** | **M** | **M** | **M** | **Moderate** | Multi-method; ICU; compliance barriers; qualitative elements informative |
| **30** | **Sujan et al. (2016)** | MMAT | **M** | **M** | **M** | **M** | **Moderate** | Sociotechnical analysis; conceptual; limited empirical grounding |
| **31** | **Magrabi et al. (2012)** | MMAT | **M** | **M** | **M** | **M** | **Moderate** | Incident analysis; FDA data; under-attribution acknowledged; classification contribution |
| **32** | **Samaranayake et al. (2012)** | MMAT | **M** | **M** | **M** | **H** | **Moderate** | 5-year incident data; single HK hospital; mechanism description strong; no incidence denominator |
| **34** | **Brown et al. (2017)** | MMAT | **M** | **L** | **M** | **M** | **Moderate** | Systematic review; typology development; broad CPOE evidence |
| **35** | **Ash et al. (2006)** | MMAT | **M** | **M** | **M** | **M** | **Moderate** | Ethnographic; 18 US sites; qualitative; transferability to other settings uncertain |
| **37** | **Ash et al. (2004)** | MMAT | **M** | **M** | **M** | **M** | **Moderate** | Qualitative; USA multi-site; foundational unintended consequences framework |
| **38** | **Graber et al. (2015)** | MMAT | **H** | **H** | **H** | **H** | **Low** | Malpractice analysis; major attribution bias; US litigation context; under-reporting likely high |
| **39** | **Ahmed et al. (2011)** | MMAT | **M** | **M** | **M** | **M** | **Moderate** | Controlled experiment; ICU; two-interface comparison; single site |
| **40** | **Middleton et al. (2013)** | MMAT | **M** | **M** | **M** | **M** | **Moderate** | Expert consensus + policy; usability framing; limited primary data |
| **41** | **Harpaz et al. (2014)** | MMAT | **M** | **M** | **M** | **M** | **Moderate** | Narrative review; text mining for ADEs; comprehensive scope; no primary data |
| **42** | **Murff et al. (2011)** | MMAT | **M** | **M** | **M** | **H** | **Moderate** | NLP validation; single US surgical site; proof-of-concept; limited transferability |
| **44** | **Nistal-Nuno (2022)** | MMAT | **M** | **M** | **M** | **H** | **Low** | Algorithm paper; online pharmacy; clinical validation absent; generalisation very limited |
| **45** | **Khairat et al. (2018)** | MMAT | **M** | **M** | **M** | **M** | **Moderate** | Critical analysis; mixed CDS adoption literature; systematic elements partial |
| **46** | **Bellini et al. (2022)** | MMAT | **M** | **M** | **M** | **M** | **Moderate** | Systematic review; perioperative ML; heterogeneous studies; external validity moderate |
| **47** | **Alghamdi (2025)** | MMAT | **L** | **M** | **M** | **H** | **Low** | Narrative review; AI drug discovery; limited medication safety relevance; governance context only |
| **48** | **Martins et al. (2018)** | MMAT | **M** | **M** | **M** | **M** | **Moderate** | Comparative; Brazilian ICU; trigger tools vs chart review; single institution |
| **49** | **Evans et al. (2001)** | MMAT | **M** | **L** | **L** | **M** | **Moderate** | PRR methodology; pharmacovigilance; foundational; limited to signal generation |
| **50** | **Topol (2019)** | MMAT | **M** | **M** | **M** | **M** | **Moderate** | High-profile narrative review; AI augmentation framing; no primary data; expert authority |
| **51** | **Wright & Sittig (2008)** | MMAT | **M** | **M** | **M** | **M** | **Moderate** | Framework analysis; CDS architecture; theoretical contribution; limited empirical testing |
| **53** | **McCoy et al. (2014)** | MMAT | **M** | **M** | **M** | **M** | **Moderate** | Review and proposal; alert appropriateness; USA-centred; no primary data |
| **54** | **Lipsitz (2012)** | MMAT | **M** | **M** | **M** | **M** | **Moderate** | Conceptual; complex systems framing; broadly applicable; no primary data |
| **55** | **Dy & Purnell (2012)** | MMAT | **M** | **M** | **M** | **M** | **Moderate** | Conceptual review; shared decision-making; indirect relevance to medication safety |
| **56** | **Steyerberg & Vergouwe (2014)** | MMAT | **L** | **L** | **L** | **M** | **High** | Methodological; clinical prediction model standards; widely adopted; foundational |
| **57** | **Holstiege et al. (2015)** | MMAT | **M** | **M** | **M** | **M** | **Moderate** | Systematic review; antibiotic prescribing CDS; moderate heterogeneity |
| **58** | **Shojania et al. (2010)** | MMAT | **M** | **M** | **M** | **M** | **Moderate** | Systematic review; point-of-care reminders; broad design types; physician performance focus |
| **59** | **Classen et al. (2011)** | MMAT | **M** | **M** | **M** | **M** | **Moderate** | Expert consensus; DDI list; limited empirical testing across institutions |
| **60** | **Phansalkar et al. (2012b)** | MMAT | **M** | **M** | **M** | **M** | **Moderate** | Expert consensus; high-priority DDI framework; practical contribution |
| **61** | **Obermeyer et al. (2019)** | MMAT | **M** | **L** | **L** | **M** | **High** | Retrospective; large US commercial dataset; strong equity finding; care-management algorithm, not CDSS directly |
| **63** | **Kiguba et al. (2023)** | MMAT | **M** | **M** | **M** | **M** | **Moderate** | Narrative review; Africa focus; LMIC pharmacovigilance; systematic elements partial |
| **67** | **Sittig & Singh (2010)** | MMAT | **M** | **M** | **M** | **M** | **Moderate** | Conceptual; sociotechnical model; broadly cited; no primary data |
| **68** | **Coiera (2015)** | MMAT | **L** | **L** | **L** | **M** | **High** | Reference textbook; comprehensive scope; authoritative; not primary research |
| **69** | **Baethge et al. (2019)** | MMAT | **L** | **L** | **L** | **L** | **High** | Methodological; SANRA validation; rigorously designed; widely applicable |
| **71** | **Bastoni et al. (2021)** | MMAT | **M** | **M** | **M** | **M** | **Moderate** | Umbrella review; eHealth implementation; dementia care; governance context |
| **73** | **Bayoumi et al. (2014)** | MMAT | **M** | **M** | **M** | **M** | **Moderate** | Population-based; Canadian older adults; medication-related ED visits; administrative data |
| **75** | **Agrawal (2009)** | MMAT | **M** | **M** | **M** | **M** | **Moderate** | Narrative review; IT for medication safety; broad scope; older evidence base |

*Rating key: L = Low risk / low concern; M = Moderate concerns / some limitations; H = High risk / major limitations; NR = Not applicable for this design/domain. Overall rating: High = predominantly low risk across domains; Moderate = some concerns in one or more domains; Low = significant limitations impeding confidence in findings. Colour coding: green = Low; amber = Moderate; red = High.*

*Abbreviations: MMAT, Mixed Methods Appraisal Tool (v2018); PRISMA, Preferred Reporting Items for Systematic Reviews and Meta-Analyses; RoB 2, Cochrane Risk of Bias 2 tool; ROBINS-I, Risk Of Bias In Non-randomised Studies of Interventions.*
